# A comprehensive meta-analysis of exogenous estrogen, progesterone, and testosterone in animal models of ischemic and hemorrhagic stroke

**DOI:** 10.1186/s13293-026-00828-6

**Published:** 2026-01-29

**Authors:** Tiffany F. C. Kung, Angely Claire C. Suerte, Elmira Khiabani, Marin Parranto, Sara Gannon Arnott, Anna C. J. Kalisvaart, Shinichi Nakagawa, Ana C. Klahr, Frederick Colbourne

**Affiliations:** 1 Department of Psychology, University of Alberta, T6G 2E9 Edmonton, AB Canada; 2 Neuroscience and Mental Health Institute, University of Alberta, Edmonton, AB Canada; 3 Department of Biological Sciences, University of Alberta, Edmonton, AB Canada; 4 Department of Social Sciences, Augustana Faculty, University of Alberta, Camrose, Canada; 5 Women and Children’s Health Research Institute, University of Alberta, Edmonton, Canada

**Keywords:** Ischemic stroke, Intracerebral hemorrhage, Subarachnoid hemorrhage, Estrogen, Progesterone, Testosterone, Pre-Clinical Meta-Analysis, Animal research

## Abstract

**Background:**

Exogenous sex hormones have been extensively studied for their influence on stroke risk and outcome. This meta-analysis served to update the pre-clinical acute ischemic stroke (AIS) literature and provide the first synthesis of the intracerebral hemorrhage (ICH) and subarachnoid hemorrhage (SAH) literature on how estrogen, progesterone, and testosterone affect post-stroke outcomes.

**Methods:**

This study was pre-registered with PROSPERO (CRD42024544794). Medline, EMBASE, Scopus, and Web of Science were searched; studies using animal models of stroke investigating exogenous estrogen, progesterone, or testosterone, alone or in combination, compared to non-treated controls were included. Assessments of injury volume, edema, and behaviour (neurological deficits, sensorimotor and cognitive outcomes) were analyzed via hierarchical meta-analyses. Risk of bias was assessed via SYRCLE and CAMARADES, and evidence certainty via an adaptation of the GRADE tool.

**Results:**

In total, 211 studies were included. Estrogen and progesterone improved all post-AIS outcomes (SMDs = 0.32–1.30, 95% CIs [0.02, 2.07], very low to moderate certainty of evidence), whereas testosterone had mostly null effects (very low to moderate certainty). Fewer studies investigated hemorrhagic stroke, with null effects of estrogen (very low to low certainty) and conflicting results of progesterone (SMDs = 0.15–1.16 [-2.20, 2.58], very low to moderate certainty) in ICH, as well as benefit of progesterone in SAH (SMD = 2.63 [0.98, 4.30], very low certainty). Uncertainty in our evidence arose from low scientific and translational rigor. Sex and gonadal status were consistent moderators of these effects, and gonadal depletion length (i.e., the ‘timing hypothesis’) was a significant moderator of estrogen’s effect on post-AIS injury volume.

**Conclusions:**

Estrogen and progesterone are promising cerebroprotectants for AIS. Further focussed and rigorous pre-clinical research on remaining research gaps (e.g., dosage parameters) are needed to guide clinical investigations and maximize the likelihood of translational success. The impact of testosterone and sex hormones in hemorrhagic stroke remain inconclusive due to lack of research.

**Supplementary Information:**

The online version contains supplementary material available at 10.1186/s13293-026-00828-6.

## Highlights

Exogenous sex hormones (estrogen, progesterone, testosterone) have been linked to increased stroke risk in humans and improved post-stroke outcomes (e.g., motor and cognitive functions) in animals.

This pre-clinical meta-analysis included experimental studies investigating exogenous estrogen, progesterone, and/or testosterone in animal models of ischemic stroke, intracerebral hemorrhage (ICH), or subarachnoid hemorrhage (SAH).

Estrogen and progesterone are cytoprotective following ischemic stroke, though further rigorous research on key issues (e.g., dosage parameters) are needed prior to clinical trials.

Sex, gonadal status, and related factors (e.g., length of gonadal depletion, or the ‘timing hypothesis’) are moderators of sex hormone effects.

The effect of testosterone in AIS and all sex hormones in hemorrhagic stroke are unclear due to lack of research.

**Supplementary Information:**

The online version contains supplementary material available at 10.1186/s13293-026-00828-6.

## Introduction

Sex hormones like estrogen, progesterone, and testosterone have been widely studied in humans and animals for their impact on acute ischemic stroke (AIS), intracerebral hemorrhage (ICH), and subarachnoid hemorrhage (SAH). Clinically, long-term exogenous estrogen and progesterone are associated with increased stroke risk across age and gonadal status [1–3], whereas the associated risk of exogenous testosterone use is less clear [3]. Pre-clinically, estrogen and progesterone are often cytoprotective, whereas testosterone is reported to be harmful [4–7]. As neurosteroids, sex hormones have been reported to influence injury and recovery via multiple avenues [7–9]. Mechanistically, estrogen, progesterone, and testosterone act upon inflammation, oxidative stress, and cell death [7–9]. These effects are predominantly mediated through classical estrogen, progesterone, and androgen receptors (ERs, PRs, and ARs), though research has also suggested the involvement of non-classical G-protein coupled receptors and transmembrane receptors [7, 8, 10].

Sex hormone effects on stroke risk and outcome are modulated by factors related to sex and gonadal status [1, 4]. For example, initiating exogenous estrogen early following gonadal hormone depletion (e.g., menopause, ovariectomy (OVX)) results in less harmful effects on stroke risk clinically, and greater benefit on stroke outcomes pre-clinically, a phenomenon known as the ‘timing hypothesis’ [1, 4, 11]. Accordingly, clinical recommendations have been shifting to encourage initiation of menopausal hormone replacement therapy (HRT) at younger perimenopausal ages [12]. As such, studying the effects of exogenous hormones in animals can provide valuable insight into mechanistic considerations and post-stroke management in patients taking exogenous sex hormone regimens such as HRT, gender affirming hormone therapy, or oral contraceptives. Further, it may be additionally possible to harness the cytoprotective effects of estrogen and progesterone in a broader stroke population without triggering their harmful effects on stroke risk. Indeed, clinical studies mostly investigate female patients prescribed HRT initiated in a delayed manner following menopause [1, 11], while the pre-clinical literature reports benefit of sex hormones when administered in male and female rodents soon after gonadal depletion [4–6].

Overall, translation rates in the stroke field remain low, due to deficits in study rigor, and a lack of collaboration across the translational pipeline [13–16]. Notably, previous research suggests that animal data has minimal influence on which therapies enter clinical trials and how they are investigated, and that this contributes to translational failures [14, 17, 18]. Pre-clinical meta-analyses may aid in bridging this gap by summarizing and evaluating the existing data on cytoprotective therapies, providing concise and comprehensive overview of literature bases in an era of ‘literature overload’ [19]. This pre-clinical meta-analysis provides an update of the literature on estrogen and progesterone in AIS (most recent meta-analyses in 2014 [6] and 2013 [5], respectively) using statistically more rigorous techniques [20–22] and evidence certainty grading [22]. This is also the first meta-analysis of the literature on estrogen and progesterone combination (COMBO) treatments and testosterone in AIS, as well as on sex hormone therapies in ICH and SAH. We examine whether the existing animal data provides consistent evidence of estrogen and progesterone’s benefit, how testosterone may affect post-stroke outcomes, and identify remaining research gaps in the animal data that will further aid in guiding both pre-clinical and clinical efforts.

## Methods & Materials

This meta-analysis was pre-registered with PROSPERO (CRD42024544794) [23]. Briefly, animal models of stroke were included, while tangential models (e.g., iron injection) were excluded. Administration of exogenous estrogen, progesterone, testosterone, its metabolites (e.g., dihydrotestosterone), and clinical formulations (e.g., Premarin), either alone or in combination with each other, were included. Phytohormones, selective receptor modulators, and precursors (e.g., pregnane) were excluded. Dissimilar to our pre-registration, combination treatments with other drugs and regimens were excluded due to a lack of research on each combination; only combination treatments with tPA or rehabilitation were included for their translational relevance. Outcomes reported more than 1 hour post-hormone administration were included. This manuscript is reported according to PRISMA guidelines [24], and a completed checklist is available as supplemental material (S1). All raw data (extraction files, R code) are available on the Open Science Framework (https://osf.io/kf7t2/overview).

### Search terms

Medline, EMBASE, Scopus, and Web of Science were searched on May 30, 2024 and again on May 1, 2025. A total of seven search terms were formulated: one per stroke subtype and sex hormone, and one limiting results to animal data (S2). References were also gathered from previous reviews [4–7].

### Screening and extraction

Abstracts and full-texts were independently screened by two reviewers via the Covidence platform [25], and a third reviewer was consulted for disagreements. Data for each study was independently extracted by two reviewers using a combination of Covidence and Excel, and disagreements were resolved by re-review of the original article and discussion. Due to the volume of studies captured, authors were only contacted when full-texts could not be retrieved. We did not receive any responses from those contacted.

Study (e.g., author), animal (e.g., comorbidities), stroke (e.g., model), intervention (e.g., formulation), and outcome (e.g., timepoint) details were extracted for data synthesis. Only the last (temporally) or maximal (spatially) assessment of injury volume, our primary outcome, was included. Edema assessed as early as 12 h post-stroke onset was included, and only the maximal score was extracted. For behavioural outcomes (neurological deficits, sensorimotor and cognitive outcomes), all early (< 7 days) assessments were extracted, as was the latest (≥ 7 days) assessment. Further details are in our pre-registration [23].

### Study quality and risk of bias

Study quality and risk of bias were independently assessed by two authors using the CAMARADES [26] checklist and the SYRCLE [27] tool. These were supplemented with a custom scale that assessed whether studies specified primary outcomes and checked assumptions of parametric statistics. Disagreements were settled by a re-review of the original article and discussion. Evidence certainty was graded via an adaptation of the GRADE guidelines, using standardized mean differences (SMD) = 0.26 as the minimally important difference and 241 subjects as the optimal information size (for further details, see S2) [22, 28, 29].

### Statistical analysis

A detailed account of our analysis is provided in the supplemental material (S2). Our code was adapted from published guides [20, 21]. All analysis was conducted using R studio [30] and its packages [31–37]. Data were converted to parametric summary statistics [38] and missing standard deviations were imputed as needed [34]. SMDs were estimated by Hedge’s G and are reported with 95% confidence/prediction intervals (CI/PI [21, 39]), with significance set at α = 0.05; positive values favoured treatment and negative values favoured control. In all instances, the simplest and best-fitting meta-analytic model (hierarchical [20, 21] or cross-classified [40]) was applied. Model robustness, or whether model results were dependent on the correlation (ρ = 0.5) we arbitrarily specified for our variance-covariance matrix, was checked using either robust variance estimation [41] or by setting a higher ρ for the matrix [20, 21]. Heterogeneity was calculated using the I^2^ and τ^2^ statistics, and publication bias via the small study effect and decline effect [21]. Mortality rates were calculated as risk ratios. Meta-regression was explored where possible. The results of intercept-removed (subgroups compared against the overall meta-analytic estimate) and intercept-included (subgroup compared against one another) models are reported; these concepts are discussed in further detail elsewhere [21, 31]. Additional moderators were investigated in large datasets via model averaging and are reported in detail in the supplemental material (S3). Importantly, these findings are exploratory and thus prone to Type I error [21]. Sensitivity analyses [42] were conducted when unrealistic and outlying SMDs (e.g., > 3000) were observed. The full-dataset model results are reported within the text, while the outlier-removed datasets are plotted in the figures to aid in data visualization. No sensitivity analyses changed our conclusions. The results of all meta-analysis and meta-regressions are available in the supplemental material (Table S3.1).

## Results

### Search results

**Fig. 1:**
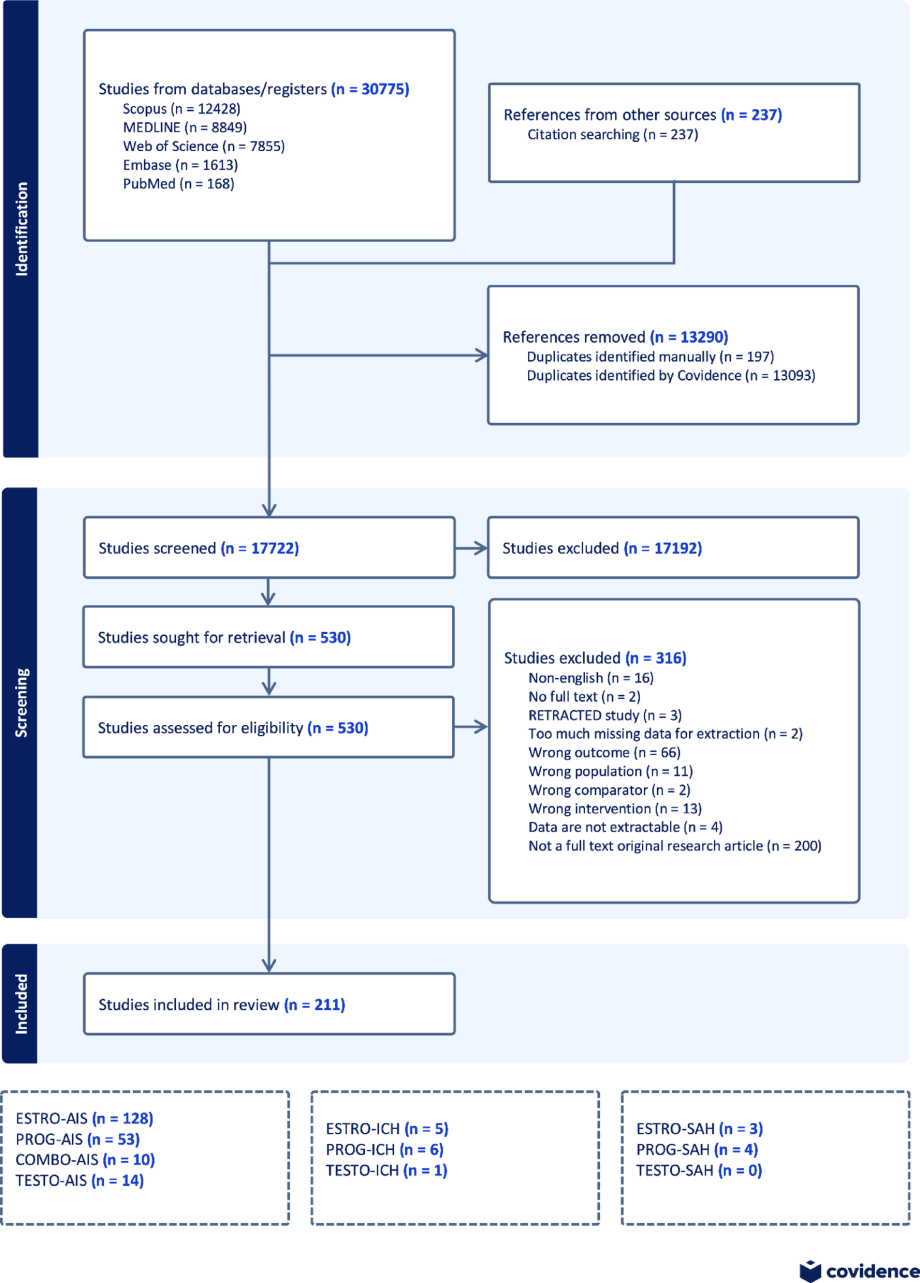
**PRISMA Flowchart**. Our search of Medline, EMBASE, Scopus, and Web of Science captured 17,722 eligible abstracts. Of these, 530 full-texts were screened. Ultimately, 211 full-texts were included: 128 on estrogen in AIS, 53 on progesterone in AIS, 10 on COMBO in AIS, 5 on estrogen in ICH, 6 on progesterone in ICH, 1 on testosterone in ICH, 3 on estrogen in SAH, and 4 on progesterone in SAH. ESTRO = estrogen, PROG = progesterone, COMBO = estrogen + progesterone combination treatment, TESTO = testosterone, AIS = acute ischemic stroke, ICH = intracerebral hemorrhage, SAH = subarachnoid hemorrhage

We identified 17,722 potential studies; 17,192 abstracts and 530 full-texts were screened, and 211 eligible full-texts were included (Fig. 1) [43–253]. Included studies are listed in S3 and detailed in Table S3.2. Only endpoints with sufficient data to meta-analyze (i.e., *n* ≥ 3 studies) are discussed; other results are provided in S3.

### Estrogen improved post-AIS outcomes

#### Injury volume

Estrogen significantly and robustly reduced injury volume (SMD = 0.87, 95% CI [0.73, 1.01], 95% PI [−0.46, 2.20], *p* < 0.001, moderate certainty). Heterogeneity was significant (*p* < 0.001). There was no evidence of a small study effect (*p* = 0.21), though there were suggestions (*p* = 0.054) of an ‘increase’ effect (i.e., effect sizes tended to increase over time [21]).

**Fig. 2:**
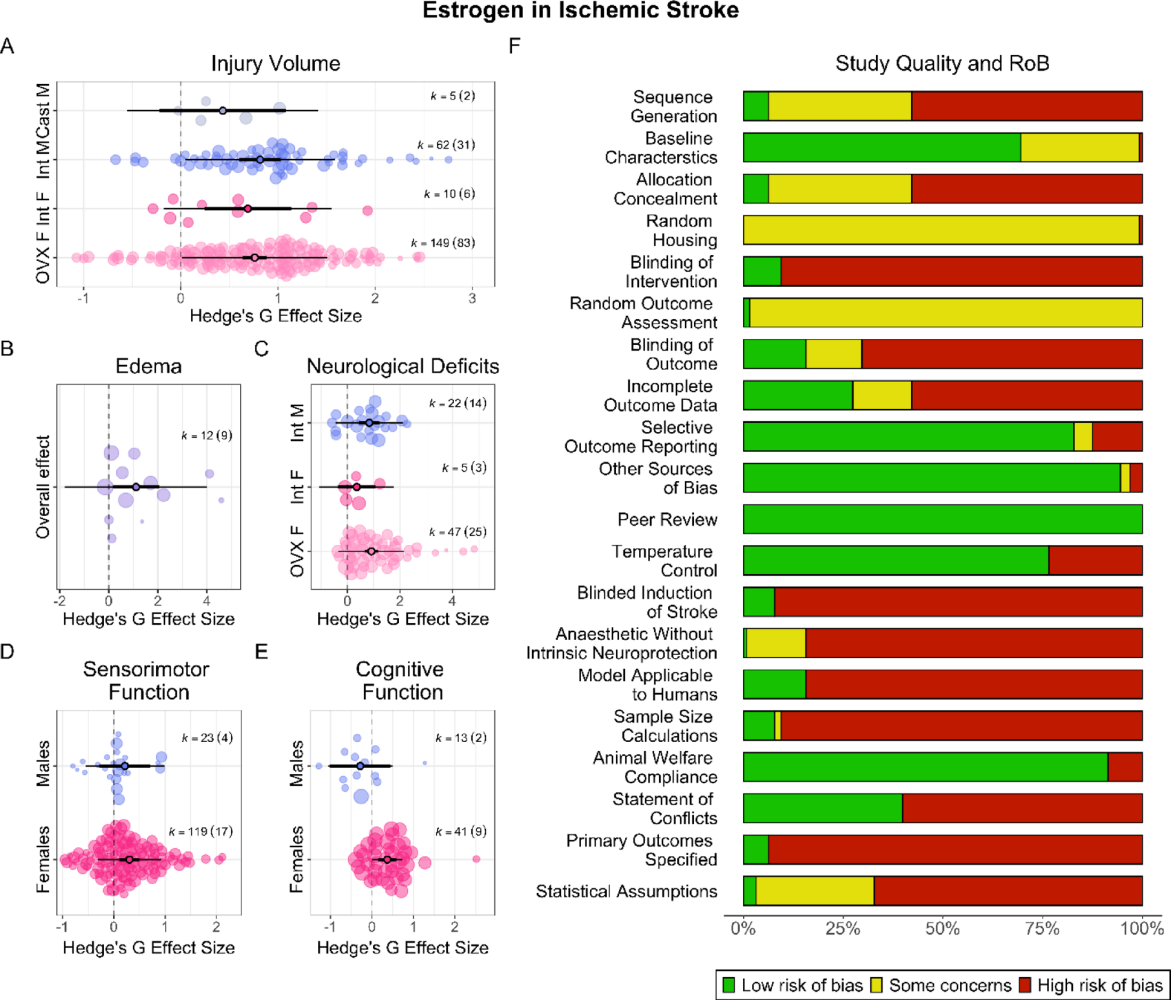
**Estrogen in AIS**. Overall, estrogen improved all post-AIS outcomes, modulated by sex and gonadal status. Estrogen reduced injury volume in females and gonadally intact males, but not in gonadally depleted males (**A**). Estrogen reduced edema (**B**), though sex and gonadal status could not be explored. Estrogen reduced neurological deficits in OVX females and gonadally intact males, but not gonadally intact females (**C**). Finally, estrogen improved sensorimotor and cognitive outcomes in females, but not in males (**D, E**). Overall, scientific and translational rigor was low (**F**). Only 58% of studies reported randomization, 16% reported blinding all extracted outcomes, and 8% of studies conducted a priori sample size calculations (median n = 7.5). Only 16% of studies employed aged animals or those with comorbidities. For all orchard plots, larger bubbles correspond to higher precision estimates. Thick lines indicate 95% CIs, while the thinner lines indicate 95% PIs. Positive values favour treatment (hormone), and negative values favour control. The number of effect sizes and studies the data are derived from are provided per estimate (k = effect sizes (studies))

Sex and gonadal status were significant (*p* < 0.001) moderators of estrogen’s overall effect on injury volume (Fig. 2 A), with an R^2^ = 0.03 (i.e., sex and gonadal status explain 3% of the variance between effect sizes), although groups (i.e., gonadally intact/depleted males/females) did not significantly differ from one another (*p* = 0.35). Gonadal depletion length was also a significant (*p* = 0.02, R^2^ = 0.047) moderator in OVX females, with estrogen efficacy decreasing as delay increased. We additionally investigated whether commercial slow release pellet usage was associated with harm, as reported [6]. Administration method was a significant (*p* < 0.001, R^2^ = 0.19) moderator, and pellets were significantly (*p* < 0.001) less effective than other methods. Regardless, estrogen significantly lessened injury with all administration methods (*p* < 0.008).

#### Edema

Estrogen significantly and robustly reduced edema (SMD = 1.11, CI [0.15, 2.07], PI [−1.79, 4.01], *p* < 0.04, low certainty; Fig. 2B). Heterogeneity was significant (*p* < 0.001). As almost all studies employed OVX females, the effect of sex and gonadal status could not be explored. There was no evidence of a small study (*p* = 0.27) or decline effect (*p* = 0.42).

#### Behaviour

Estrogen significantly and robustly improved neurological deficits (SMD = 0.84, CI [0.64, 1.04], PI [−0.38, 2.07], *p* < 0.001, moderate certainty). Heterogeneity was significant (*p* < 0.001). There was no evidence of a small study effect (*p* = 0.63), but slight indications of an increase effect (*p* = 0.07). Sex and gonadal status were significant (*p* < 0.001, R^2^ = 0.049) moderators of the overall effect (Fig. 2C), though groups did not significantly differ from one another (*p* = 0.36). Gonadal depletion length was not a significant (*p* = 0.44) moderator.

Estrogen significantly improved sensorimotor outcomes (SMD = 0.36, CI [0.02, 0.71], PI [−0.91, 1.64], *p* = 0.04, very low certainty), though this was not robust to model misspecification (*p* = 0.08). Heterogeneity was significant (*p* < 0.001). There was no significant evidence of a small study (*p* = 0.20) or decline effect (*p* = 0.68). Sex was a significant (*p* = 0.02, R^2^ = 0.00018) moderator (Fig. 2D), though groups (i.e., males and females) did not significantly differ from one another (*p* = 0.94). Gonadal depletion length was not a significant (*p* = 0.37) moderator.

Estrogen significantly and robustly improved cognitive outcomes (SMD = 0.32, CI [0.10, 0.53], PI [−0.05, 0.68], *p* < 0.017, very low certainty). Heterogeneity was not significant (*p* = 0.24). There was no evidence of a small study (*p* = 0.92) or decline effect (*p* = 0.66). Sex was a significant (*p* = 0.005, R^2^ = 0.80) moderator (Fig. 2E), though groups did not significantly differ from one another (*p* = 0.09). Interestingly, though non-significant, the effect of estrogen on cognitive outcomes in males was slightly harmful. However, these data were based on limited findings, with almost all effect sizes (12 of 13) arising from a single study. Thus, further research on this endpoint is needed before concrete conclusions can be drawn regarding the effect of estrogen on cognitive outcomes in males. Finally, gonadal depletion length was not a significant (*p* = 0.25) moderator.

#### Study quality and risk of bias

Overall, only 42% and 16% reported randomization and blinding of all extracted outcomes, respectively (Fig. 2F). Only 10% of studies employed aged animals, 3% used diabetic animals, and 2% hypertensive animals. In total, 8% of studies reported a priori sample size calculations, netting required sample sizes between 3 and 40 (median = 7.5) per group.

### Progesterone improved post-AIS outcomes

#### Injury volume

**Fig. 3:**
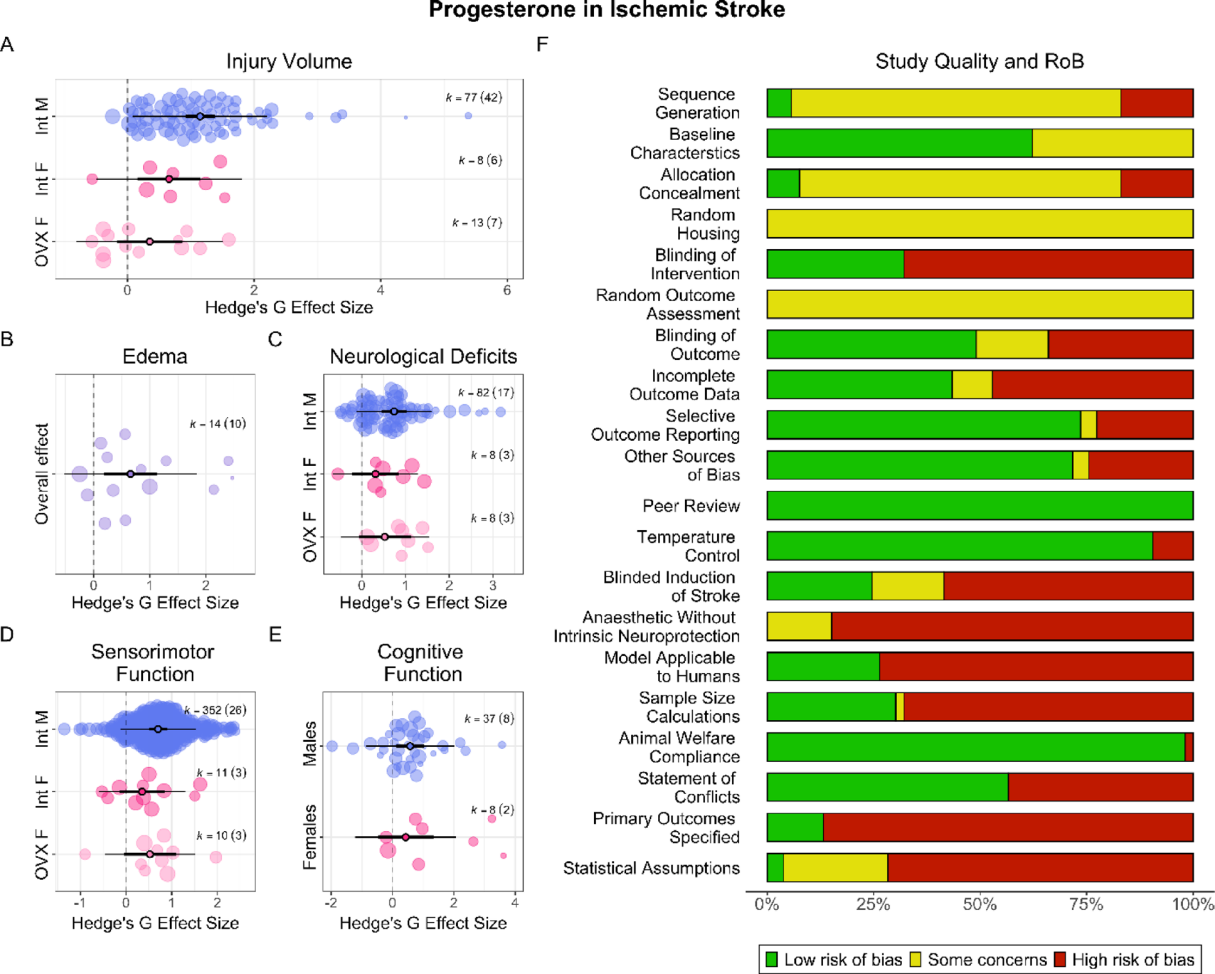
**Progesterone in AIS**. Overall, progesterone improved post-AIS outcomes, modulated by sex and gonadal status. Progesterone reduced injury volume in gonadally intact males and females, but not in OVX females (**A**). Progesterone reduced edema (**B**), but the role of sex and gonadal status could not be explored. Progesterone improved neurological deficits in gonadally intact males, but not in females, regardless of gonadal status (**C**). Progesterone improved sensorimotor outcomes in gonadally intact males, but not in females, regardless of gonadal status (**D**). Finally, progesterone improved cognitive outcomes in males, but not females (**E**). Scientific and translational rigor of included studies was mixed (**F**). While 83% of studies reported randomization, only 49% reported blinding all outcomes; 26% of studies conducted a priori sample size calculations (median n = 8). Only 26% used aged animals or those with comorbidities

Progesterone significantly and robustly reduced injury volume (SMD = 1.01, CI [0.79, 1.23], PI [−0.13, 2.15], *p* < 0.001, very low certainty). Heterogeneity was significant (*p* < 0.001). There was significant evidence of both small study (*p* = 0.04; i.e., smaller studies reported larger effect sizes [20]) and increase (*p* = 0.02) effects. When corrected for publication bias, progesterone no longer affected injury (SMD = 0.45, CI [−0.11, 1.00], *p* = 0.11). Sex and gonadal status were significant (*p* < 0.001, R^2^ = 0.24) moderators (Fig. 3A), and groups differed significantly (*p* = 0.01), with gonadally intact males benefiting from progesterone significantly (*p* = 0.006) more than ovariectomized females, with a similar non-significant trend (*p* = 0.06) observed compared to gonadally intact females. OVX and gonadally intact females were not significantly (*p* = 0.36) different.

#### Edema

Progesterone significantly and robustly reduced edema (SMD = 0.66, CI [0.19, 1.13], PI [−0.52, 1.84], *p* < 0.02, very low certainty; Fig. 3B). Heterogeneity was significant (*p* = 0.03). As almost all studies employed gonadally intact males, the effect of sex and gonadal status could not be explored. There was significant evidence of a small study effect (*p* = 0.04), but not of a decline effect (*p* = 0.63). When corrected for publication bias, progesterone no longer reduced edema (SMD = −0.82, CI [−2.31, 0.68], *p* = 0.24).

#### Behaviour

Progesterone significantly and robustly improved neurological deficits (SMD = 0.66, CI [0.42, 0.91], PI [−0.09, 1.42], *p* < 0.001, moderate certainty). Heterogeneity was not significant (*p* = 0.56). There was no evidence of a small study (*p* = 0.83) or decline effect (*p* = 0.18). Sex and gonadal status were significant (*p* < 0.001, R^2^ = 0.09) moderators (Fig. 3C), though groups did not significantly differ from one another (*p* = 0.28).

Progesterone significantly and robustly improved sensorimotor outcomes (SMD = 0.57, CI [0.23, 0.91], PI [−1.07, 2.22], *p* < 0.02, very low certainty). Heterogeneity was significant (*p* < 0.001). There was no evidence of a small study (*p* = 0.48) or decline effect (*p* = 0.12). Sex and gonadal status were significant (*p* = 0.006, R^2^ = 0.01) moderators (Fig. 3D), though groups did not differ significantly from one another (*p* = 0.25).

Progesterone significantly and robustly improved cognitive outcomes (SMD = 0.54, CI [0.14, 0.94], PI [−0.83, 1.91], *p* < 0.03, moderate certainty). Heterogeneity was significant (*p* < 0.001). There was no evidence of a small study (*p* = 0.81) or decline effect (*p* = 0.97). Sex was a significant (*p* = 0.04, R^2^ = 0.007) moderator (Fig. 3E), though groups did not differ significantly from one another (*p* = 0.76).

#### Study quality and risk of bias

In total, 83% of studies reported randomization and 49% reported blinding all outcomes (Fig. 3F). Only 21% of studies used aged animals and 8% used hypertensive animals; the rest employed young, healthy animals. Only 26% of studies conducted a priori sample size calculations, netting required group sizes of *n* = 3–15 (median *n* = 8).

#### Mortality rates

Only 40% of studies explicitly reported mortalities or lack thereof, and 32% could be meta-analyzed. Mortality rates were not significantly different between groups (RR = 1.19, CI [0.80, 1.77], *p* = 0.39).

### COMBO improved Post-AIS outcomes

#### Injury volume

**Fig. 4:**
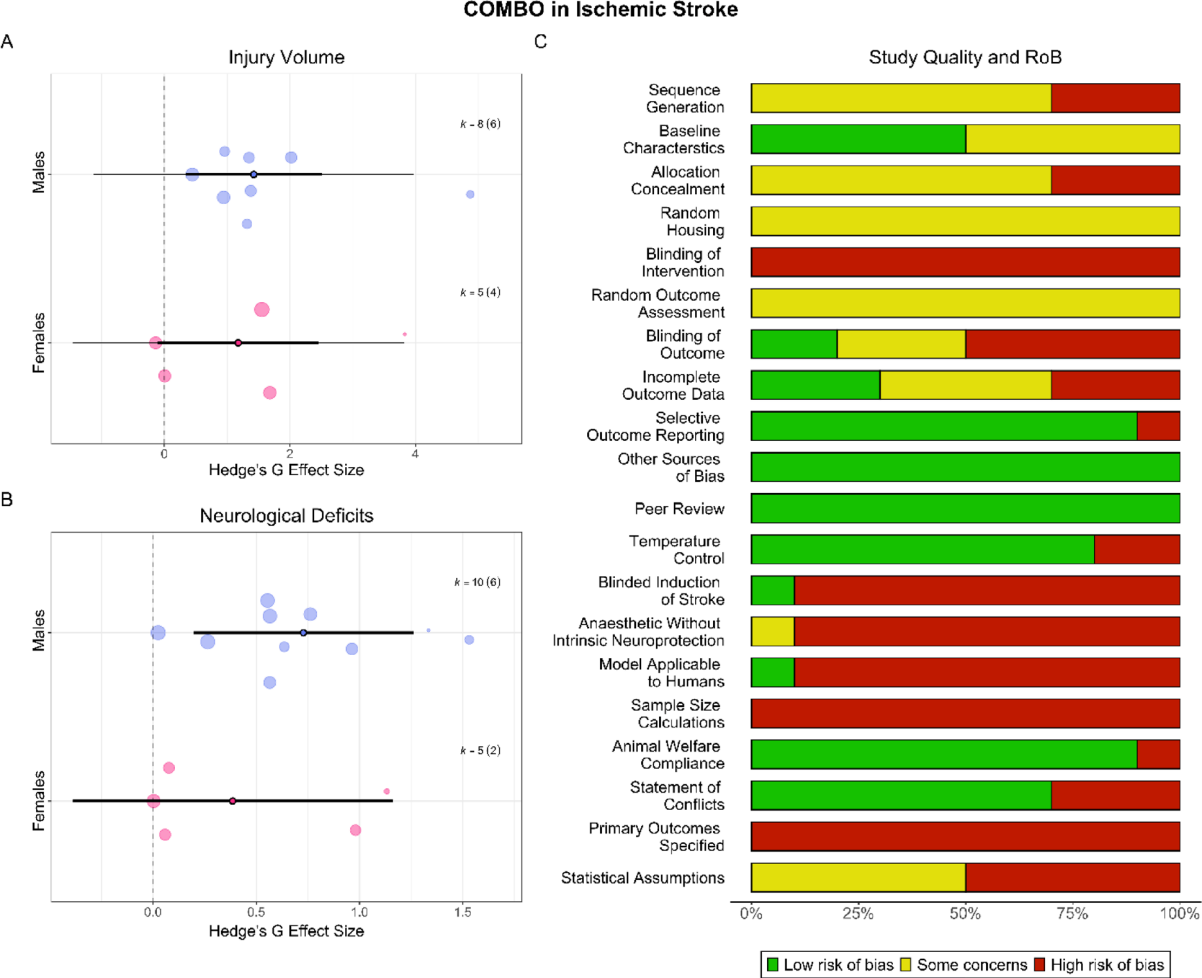
**COMBO in AIS**. A combination treatment of estrogen and progesterone (COMBO) improved injury volume (**A**) and neurological deficit (**B**) in males, but not females after AIS. Scientific and translational rigor of included studies was low (**C**). While 70% of studies reported randomization, only 20% reported blinding all outcomes, and no studies conducted a priori sample size calculations. Only one study (10%) used aged animals

COMBO significantly and robustly reduced injury volume (SMD = 1.30, CI [0.46, 2.14], PI [−1.12, 3.72], *p* < 0.01, moderate certainty). Heterogeneity was significant (*p* < 0.001). There was no evidence of a small study (*p* = 0.51) or decline effect (*p* = 0.21). COMBO was not more effective than estrogen- or progesterone-only groups (*p* > 0.70). Sex was a significant (*p* = 0.02, R^2^ = 0.01) moderator (Fig. 4A), though groups did not differ significantly from one another (*p* = 0.75).

#### Neurological deficits

COMBO significantly and robustly reduced neurological deficits (SMD = 0.62, CI [0.17, 1.06], PI [0.12, 1.12], *p* = 0.02, low certainty). Heterogeneity was not significant (*p* = 0.89). There was no evidence of a small study (*p* = 0.69) or decline effect (*p* = 0.59). Individual studies reported that COMBO was not more effective than estrogen- or progesterone-only groups, which could not be explored via meta-regression due to insufficient studies per subgroup. Sex was a significant (*p* = 0.03, R^2^ = 0.99) moderator (Fig. 4B), though groups did not differ significantly from one another (*p* = 0.45).

#### Study quality and risk of bias

Overall, 70% of studies randomized groups, but only 20% reported blinding all outcomes (Fig. 4C). All studies used young, healthy animals but one, which used aged animals. No studies conducted sample size calculations.

### Testosterone had mixed effects on post-AIS outcomes

#### Injury volume

**Fig. 5:**
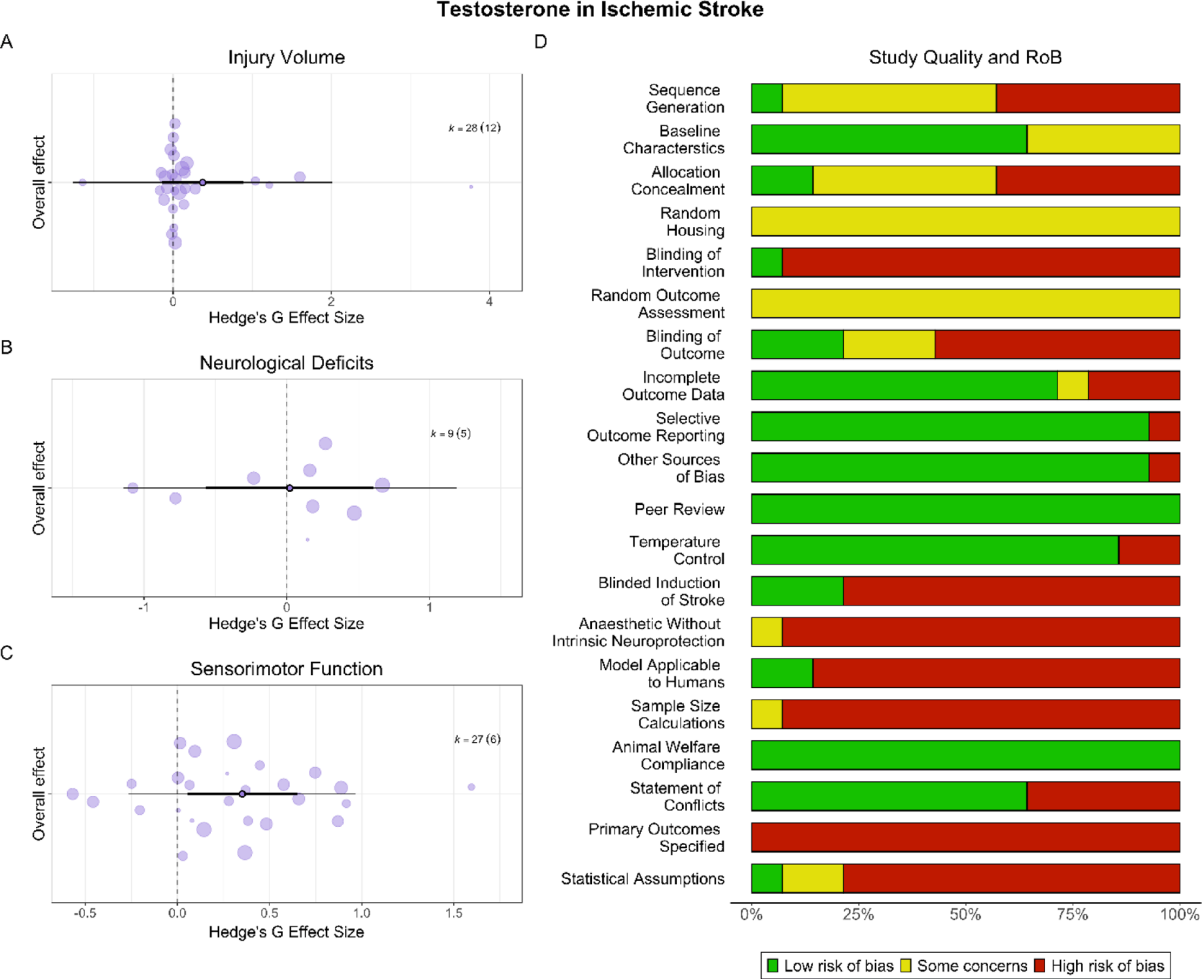
**Testosterone in AIS**. Testosterone had no effect on injury volume (**A**) or neurological deficits (**B**), but significantly improved sensorimotor outcomes (**C**) in AIS. Scientific rigor was mixed, and translational rigor was low (**D**). In total, 57% of studies reported randomization, 21% reported blinding all outcome assessments, and no studies conducted a priori sample size calculations. Only two (14%) studies used aged animals

Testosterone did not significantly affect injury (SMD = 0.37, CI [−0.14, 0.89], PI [−1.27, 2.02], *p* = 0.14, low certainty; Fig. 5A). Heterogeneity was significant (*p* = 0.009). There was no evidence of a small study (*p* = 0.65) or decline effect (*p* = 0.60). Gonadal status was not a significant (*p* = 0.41) moderator; all but one study employed male animals, so the role of sex could not be explored.

#### Behaviour

Testosterone did not significantly affect neurological deficits (SMD = 0.02, CI [−0.57, 0.61], PI [−1.15, 1.19], *p* = 0.94, very low certainty; Fig. 5B). Heterogeneity was not significant (*p* = 0.16). As all but one study employed castrated males, the role of sex and gonadal status could not be explored. There was no evidence of a small study effect (*p* = 0.69), but there was significant evidence of a decline effect (*p* = 0.04). When corrected for publication bias, testosterone still did not affect neurological deficits (SMD = 0.26, CI [−1.71, 2.22], *p* = 0.77).

Testosterone significantly and robustly improved sensorimotor outcomes (SMD = 0.65, CI [0.09, 1.21], PI [−1.68, 2.98], *p* < 0.03, moderate certainty; Fig. 5C). Heterogeneity was significant (*p* < 0.001). As all but one study employed castrated males, the role of sex and gonadal status could not be explored. There was no evidence of a small study (*p* = 0.68) or decline effect (*p* = 0.61).

#### Study quality and risk of bias

Overall, 57% of studies reported randomization, and only 21% reported blinding all outcome assessments (Fig. 5D). All studies used healthy animals, and all but two used young animals. No studies conducted a priori sample size calculations.

### Estrogen had no effect on post-ICH outcomes

#### Edema

**Fig. 6:**
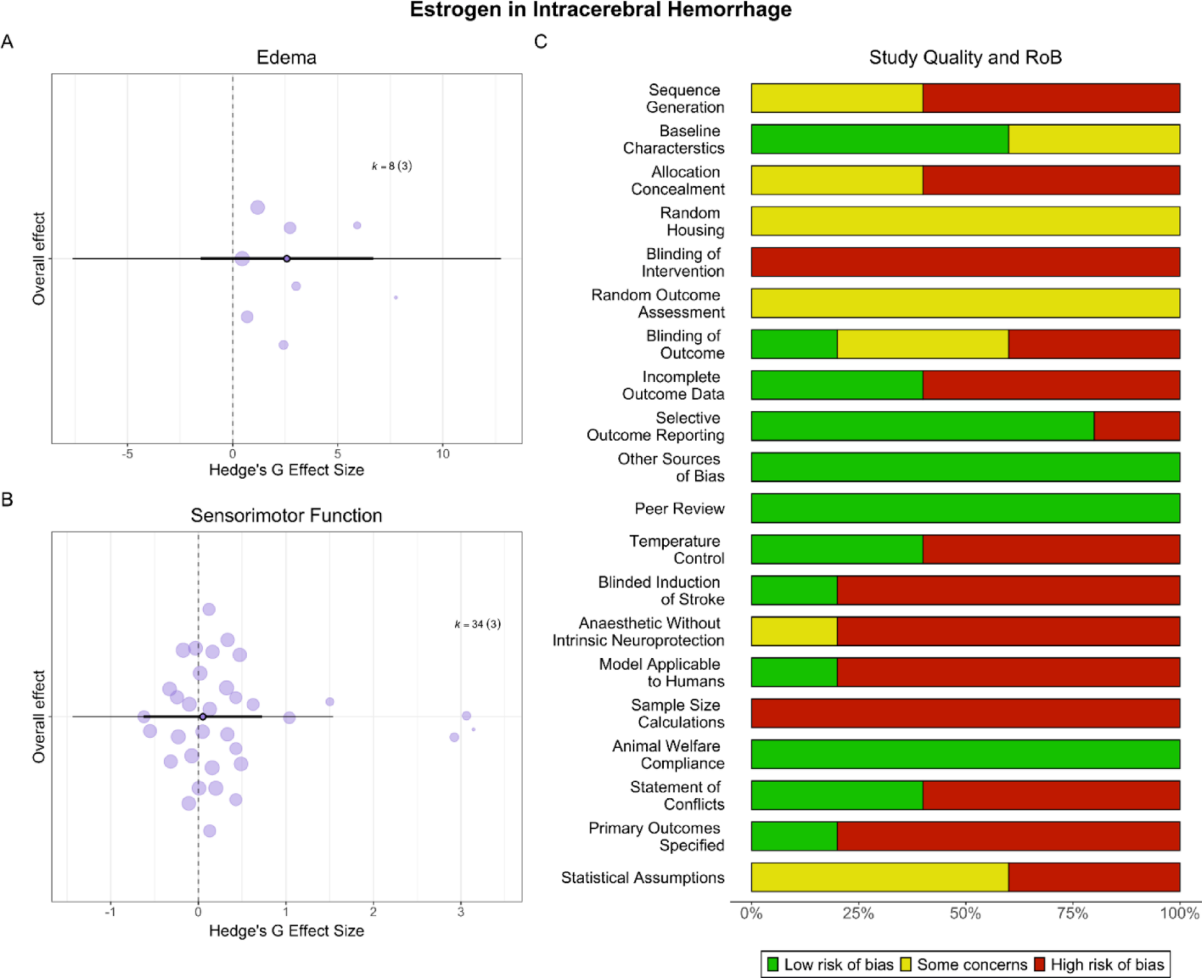
**Estrogen in ICH**. Estrogen had no statistically significant effect on edema (**A**) or sensorimotor outcomes (**B**) in ICH. Scientific and translational rigor was low (**C**); 40% of studies reported randomization, 20% reported blinding all outcomes, and no studies conducted a priori sample size calculations. Only one study (20%) used hyperglycemic males

Estrogen did not significantly affect edema (SMD = 2.58, CI [−1.53, 6.68], PI [−7.61, 12.76], *p* = 0.11, very low certainty; Fig. 6A). Heterogeneity was significant (*p* < 0.001). Meta-regressions could not be conducted due to insufficient studies per subgroup. There was no evidence of a small study (*p* = 0.91) or decline effect (*p* = 0.36).

#### Sensorimotor outcomes

Estrogen did not affect sensorimotor outcomes (SMD = 0.05, CI [−0.63, 0.73], PI [−1.44, 1.54], *p* = 0.77, low certainty; Fig. 6B). Heterogeneity was significant (*p* < 0.001). Meta-regressions could not be conducted due to insufficient studies per subgroup. There was no evidence of a small study effect (*p* = 0.15); a decline effect could not be explored.

#### Study quality and risk of bias

Overall, only 40% of studies reported randomization and 20% reported blinding all outcomes (Fig. 6C). All studies employed young, healthy animals except one, which used hyperglycemic males. No studies conducted sample size calculations.

### Progesterone had mixed effects on post-ICH outcomes

#### Injury volume

**Fig. 7:**
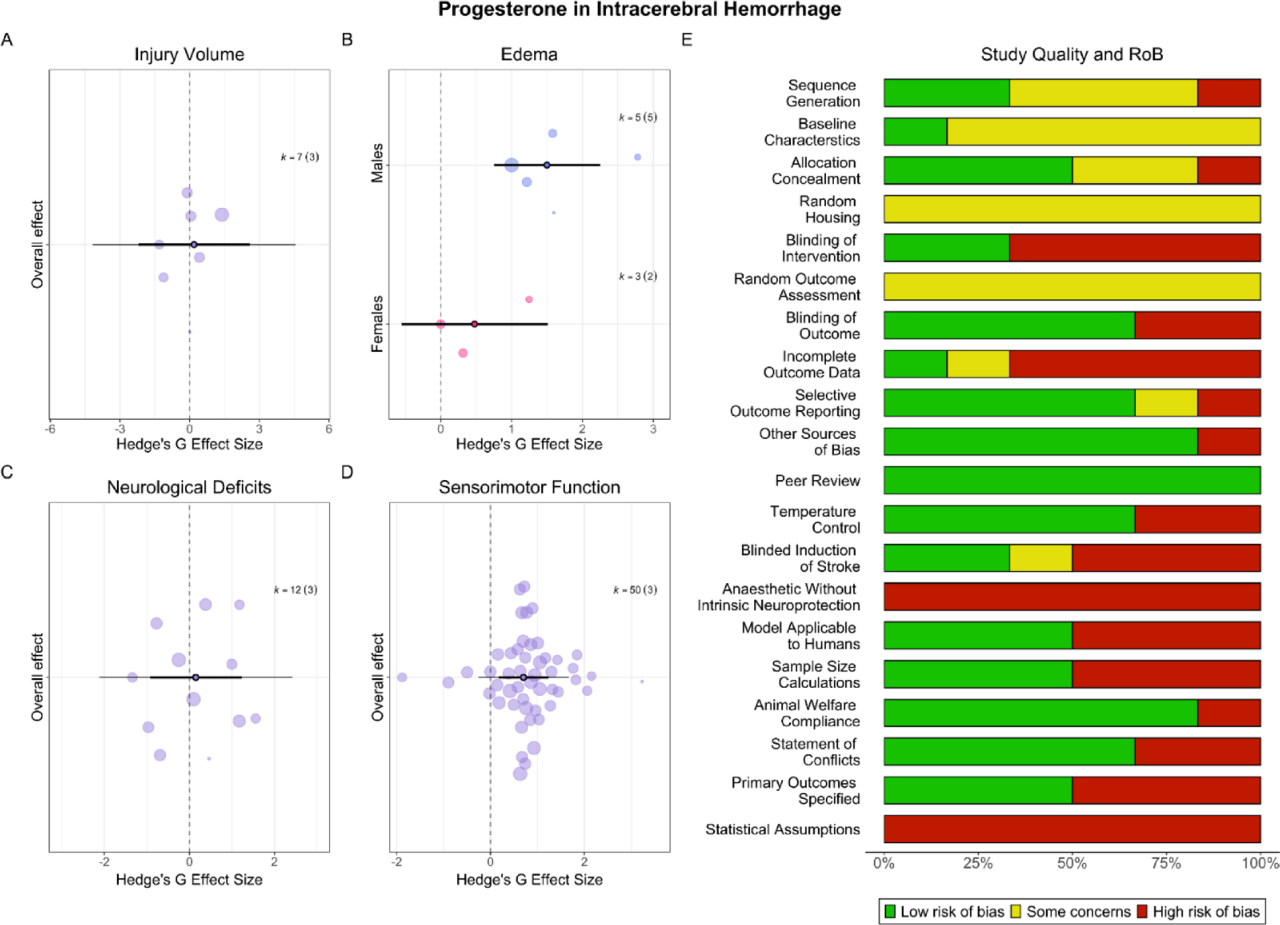
**Progesterone in ICH**. Progesterone had no effect on injury volume (**A**) in ICH. Progesterone significantly reduced edema in males, but not in females (**B**). Progesterone had no effect on neurological deficits (**C**), but significantly improved sensorimotor outcomes (**D**). Scientific and translational rigor of included studies was moderate (**E**). Overall, 83% of studies reported randomization, 67% reported blinding all outcomes, and 50% conducted a priori sample size calculations (median n = 10). In total, 50% of studies employed aged animals or those with comorbidities

Progesterone did not affect injury volume (SMD = 0.19, CI [−2.20, 2.58], PI [−4.17, 4.55], *p* = 0.77, low certainty; Fig. 7A). Heterogeneity was significant (*p* = 0.07). Meta-regressions could not be conducted due to insufficient studies per subgroup. There was no evidence of a small study effect (*p* = 0.12), and a decline effect could not be explored.

#### Edema

Progesterone significantly and robustly reduced edema (SMD = 1.16, CI [0.39, 1.92], PI [−0.04, 2.36], *p* < 0.01, moderate certainty). Heterogeneity was not significant (*p* = 0.25). There was no evidence of a small study (*p* = 0.30) or a decline effect (*p* = 0.86). Sex was a significant (*p* = 0.007, R^2^ = 0.99) moderator (Fig. 7B), though groups did not differ significantly from one another (*p* = 0.10).

#### Behaviour

Progesterone did not affect neurological deficits (SMD = 0.15, CI [−0.93, 1.23], PI [−2.12, 2.42], *p* = 0.61, very low certainty; Fig. 7C). Heterogeneity was significant (*p* = 0.002). Meta-regressions could not be conducted due to insufficient studies per subgroup. There was no evidence of a small study effect (*p* = 0.89), and a decline effect could not be assessed.

Progesterone significantly improved sensorimotor outcomes (SMD = 0.70, CI [0.17, 1.23], PI [−0.26, 1.67], *p* = 0.03, low certainty; Fig. 7D), though this was not robust to model misspecification (SMD = 0.70, CI [−0.07, 1.48], PI [−1.19, 2.60], *p* = 0.06). Heterogeneity was significant (*p* = 0.002). Meta-regressions could not be conducted due to insufficient studies per subgroup. There was no evidence of a small study effect (*p* = 0.91), and a decline effect could not be assessed.

#### Study quality and risk of bias

In total, 83% of studies reported randomization, and 67% reported blinding all outcomes (Fig. 7E). Half of studies used translationally relevant populations—two used aged animals, and one used hypertensive rats. Finally, 50% of studies conducted sample size calculations, netting required group sizes of *n* = 8–12 (median = 10).

### Progesterone may improve post-SAH outcomes

#### Neurological deficits

**Fig. 8:**
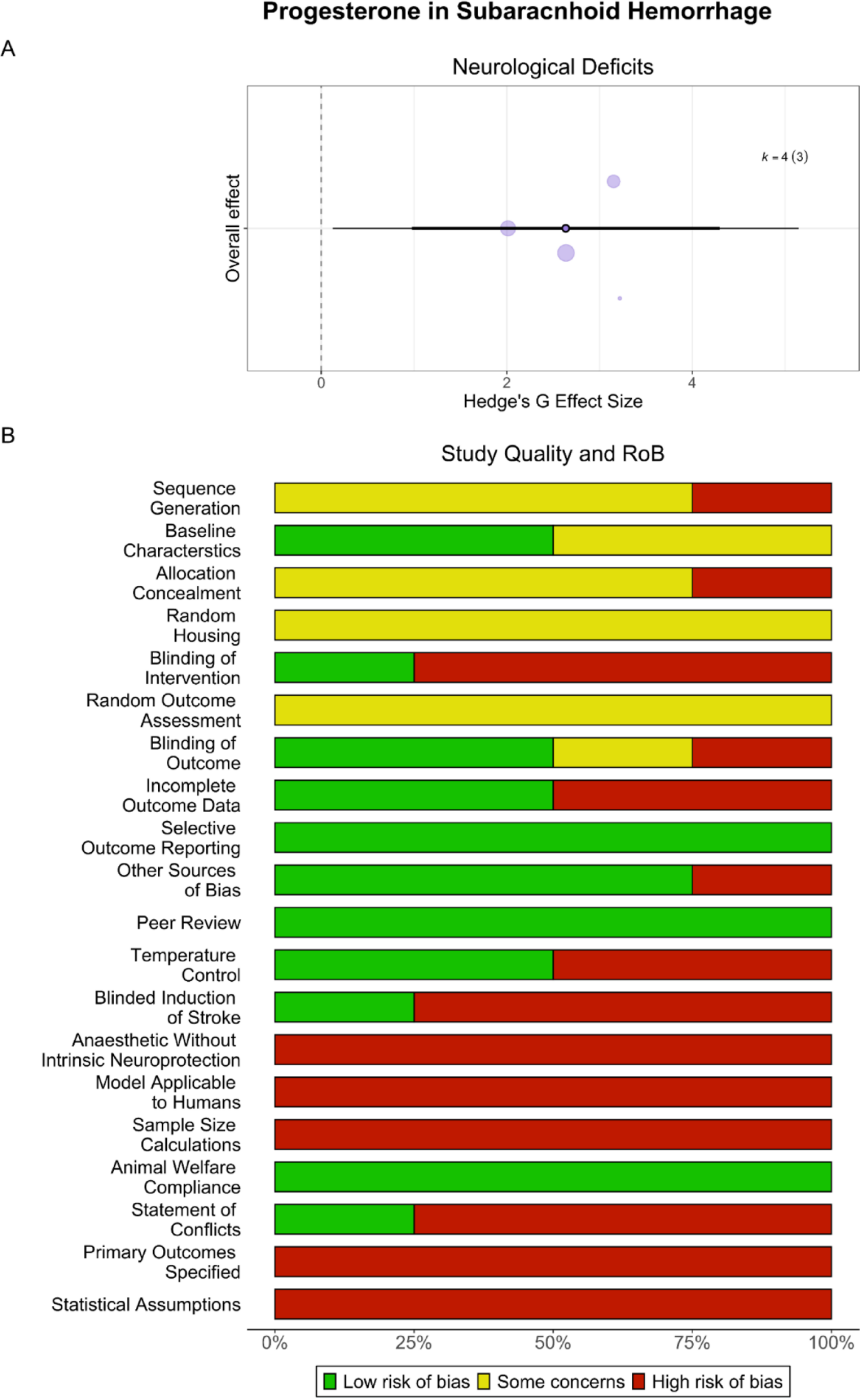
**Progesterone in SAH**. Progesterone significantly improved neurological deficits in SAH (**A**). Scientific rigor was mixed, while translational rigor was low (**B**). Overall, 75% of studies reported randomization, 50% reported blinding all outcomes, and no studies conducted a priori sample size calculations. No studies used aged animals or those with comorbidities

Progesterone significantly and robustly improved neurological deficits (SMD = 2.63, CI [0.98, 4.30], PI [0.12, 5.15], *p* < 0.02, very low certainty; Fig. 8A). Heterogeneity was not significant (*p* = 0.18). Meta-regressions could not be conducted due to insufficient studies per subgroup. There was no evidence of a small study (*p* = 0.78) or decline effect (*p* = 0.70).

#### Study quality and risk of bias

Overall, 75% of studies reported randomization, and 50% reported blinding all outcomes (Fig. 8B). All studies used young, healthy animals, and no studies conducted sample size calculations.

### Additional analyses

#### Additional potential moderators

Other potential moderators of estrogen’s effect on post-AIS outcomes were further explored via data dredging (S3). Regarding injury volume, higher dosages were associated with increased efficacy, which trended toward significance (*p* = 0.052) in the full dataset and reached significance (*p* = 0.04) in the outlier-removed dataset. In the full sensorimotor dataset, longer administration onset delays (i.e., initiation of dosing, relative to stroke and analyzed continuously), longer occlusion times, and use of the rotarod test were associated with increased estrogen efficacy (*p* ≤ 0.003), while use of the corner turn and forelimb placing tests were associated with reduced efficacy (*p* ≤ 0.008). In the outlier removed sensorimotor dataset, only age was a significant (*p* = 0.03) moderator, with increased animal age associated with reduced estrogen efficacy. No significant moderators of estrogen’s effect on neurological deficits were identified, and edema and cognitive data were not explored due to lack of research.

Model averaging was also conducted on progesterone’s effects on post-AIS outcomes. Increased age and higher dosages of progesterone were associated with reduced efficacy on injury volume (*p* ≤ 0.03), while subcutaneous administration methods were associated with greater progesterone efficacy (*p* = 0.07). In the full sensorimotor dataset, longer durations of hormone administration, later times of administration onset, longer occlusion lengths, lower dosages, earlier assessment times, and use of the grid walking or grip assessment test were all associated with increased progesterone efficacy (*p* ≤ 0.04). No significant moderators of progesterone’s effect on neurological deficits were identified, and edema and cognitive data were not explored due to lack of research. Overall, no moderators identified via data dredging were consistent across all endpoints. Further, as data dredging is prone to Type I error [21], the significance, direction, and magnitude of these results should be interpreted with caution.

#### Hormone mechanisms of action

Studies also investigated a wide diversity of mechanisms aside from those we meta-analysed, many of which overlapped across sex hormones and stroke subtypes (Tables 1 and 2). Estrogen and progesterone were reported to be anti-inflammatory and to improve blood-brain barrier integrity for AIS, ICH, and SAH. Interestingly, only 18% of studies investigating estrogen’s effects on cerebral blood flow after AIS found the hormone’s protective effects to be blood flow-dependent. Estrogen was also reported to be anti-oxidative in both AIS and ICH, and to reduce cell death in both AIS and SAH. Comparatively, progesterone administration was reported to reduce cell death and to be anti-oxidative across all stroke subtypes. Importantly, these findings were not unanimous, with some studies reporting null or aggravating effects of estrogen or progesterone on these mechanisms. Finally, studies reported testosterone to be either protective or harmful after AIS in almost equal proportion, with supposed pro- and anti- apoptotic and inflammatory actions. Interestingly, studies that reported testosterone and dihydrotestosterone to be protective tended to employ lower dosages (19–67 mg/kg) than studies that reported harm (41–192 mg/kg). No studies explicitly noted studying adverse side effects associated with hormone administration.

#### Hormone receptors

Only 16 estrogen, four progesterone, and four testosterone studies in AIS investigated whether the effects of hormone administration were receptor-mediated (Table 1). All progesterone and testosterone studies reported classical PR- or AR-dependence, and no studies explored non-classical receptors. Interestingly, one study (shown in Table 1) reported that while progesterone cytoprotection was PR-dependent, allopregnanolone, a metabolite of progesterone, had PR-independent actions. Comparatively, estrogen studies reported the hormone’s effects to be both ER-dependent and -independent, even studying the involvement of individual isoforms, ERα and ERβ. Two studies additionally reported the involvement of non-classical G-Protein Coupled Estrogen Receptors. As neurosteroids with wide and varied effects on the brain, it is unsurprising that estrogen seemingly acts via multiple receptors in the context of stroke injury [8, 10]. Likely, with further research, PR- and AR-independent actions of progesterone and testosterone, either directly or via its metabolites, will be observed as well.

#### Dosage parameters

Of the 19 estrogen in AIS [44, 52, 79, 82, 84, 98, 99, 113, 117, 127, 133, 141, 168, 175, 186, 234, 241, 242, 250] and nine progesterone in AIS [85, 166, 170, 177, 187, 197, 200, 203, 204] dose-response studies, only nine and four, respectively, investigated more than two dosages on any one of our investigated endpoints, with inconsistent trends reported. For example, seven estrogen studies reported no effect of dosage, while six favoured higher dosages, three favoured intermediate, and three favoured lower dosages.

Similarly, seven estrogen [81, 84, 93, 111, 132, 136, 219] and three progesterone in AIS [203, 204, 208] studies investigated the optimal target window and reported inconsistent trends. For example, one study reported estrogen administration to be beneficial when administered up to 3 h post-stroke [111], while another study reported no benefit of estrogen when delayed as little 0.5 h post-stroke [132]. Lastly, only 24 studies (11% of total) [45, 47, 55, 63, 70, 75, 77, 84, 88, 90, 106, 111, 119, 131, 139, 163, 166, 200, 201, 211, 220, 237, 238, 250] captured in our search investigated administration onset delays of ≥ 3 h (when patients typically arrive at the hospital [254]). Overall, no dosage range or target window clearly emerged as most efficacious.

 Only four studies directly investigated the timing hypothesis following estrogen administration in AIS [80, 103, 160, 251]. Three reported longer delays to diminish estrogen’s neuroprotective efficacy [103, 160, 251], while one reported a complicated profile that interacts with age [80]. While all of these studies assessed injury volume, only three assessed neurological deficits [80, 103, 160], and only two studies investigated sensorimotor and cognitive function [69, 160].

### Study quality

We assessed the impact of individual quality domains (e.g., randomization) on effect size inflation, as has been previously reported [26, 255, 256]. Overall, reporting of SYRCLE and CAMARADES domains have improved over time (Fig. S3.1). Randomization (*p* = 0.23), incomplete outcome reporting (*p* = 0.69), presence of other sources of bias (*p* = 0.49), and use of translationally relevant models (*p* = 0.12) were not significant moderators of the effect of sex hormones on injury volume, suggesting they were not independently associated with inflated effect sizes. A lack of blinding (*p* = 0.06) trended toward significance, with un-blinded studies tending to report larger effect sizes, as seen elsewhere [257]. Generally, studies that were blinded reported smaller (SMD = 0.22, 95% CI [−0.01, 0.46]) effect sizes than studies that were not (SMD = 0.73, CI [0.60, 0.86]).

## Discussion

**Fig. 9:**
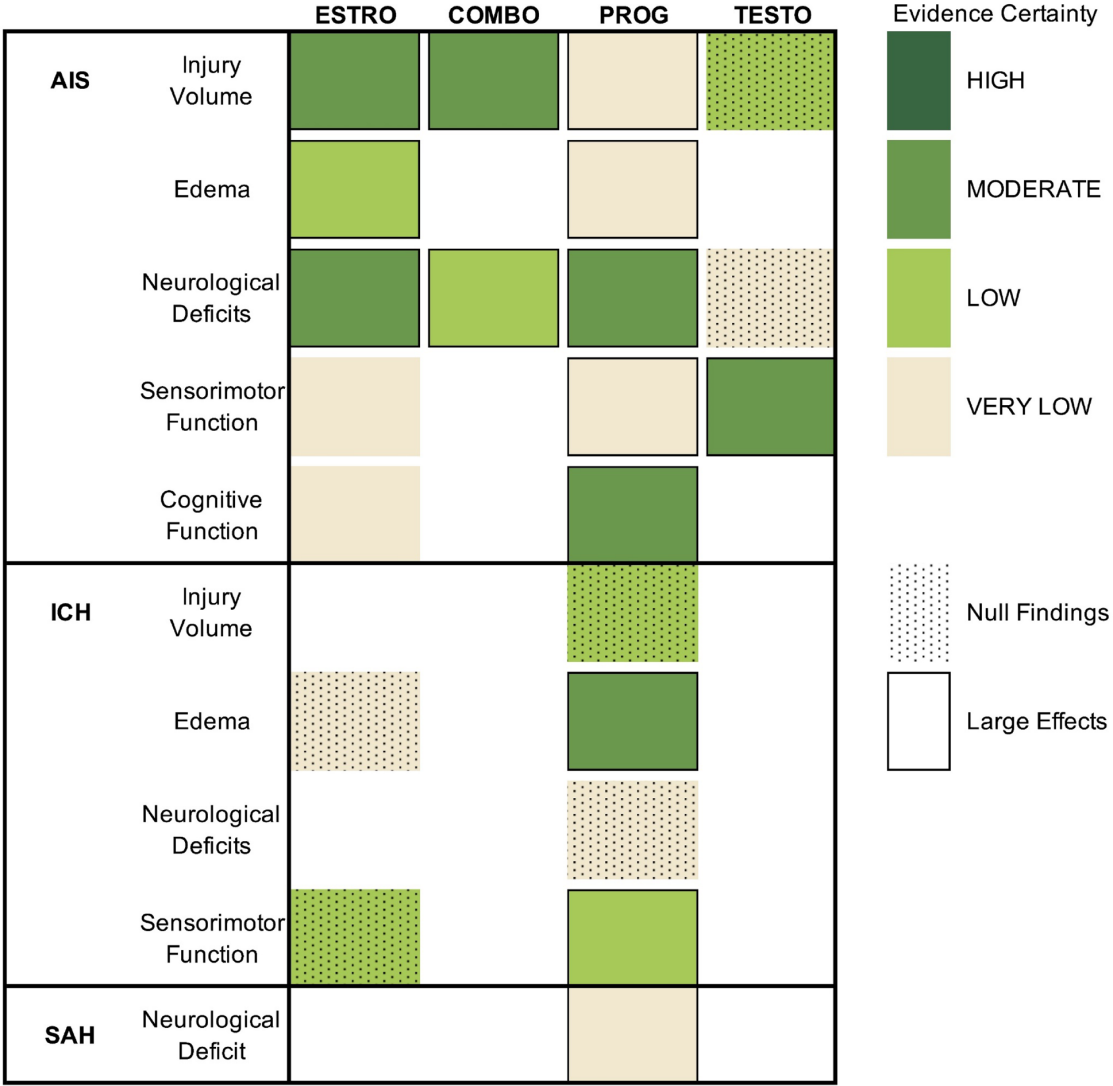
**Main Findings**. The certainty of the evidence on all endpoints is provided. A benchmark of SMD ≥ 0.49 was set for large effects. Estrogen and progesterone improved all post-AIS outcomes, with varying degrees of certainty. Comparatively, estrogen had null effects and progesterone had conflicted effects on post-ICH outcomes. Progesterone improved post-SAH neurological deficits. Finally, testosterone had conflicted effects on post-AIS outcomes, with mostly null effects, though the certainty of the evidence was low

Our meta-analysis provides a comprehensive and rigorous overview of the exogenous sex hormone literature in all three subtypes of experimental stroke (Fig. 9). Estrogen, progesterone, and COMBO significantly improved all post-AIS outcomes (SMDs = 0.32–1.30, very low to moderate certainty). Comparatively, the effects of testosterone were conflicted (SMDs = 0.02–0.65, very low to moderate certainty). Due to the scarcity of studies, not all sex hormones could be analyzed following ICH and SAH. Estrogen had null effects on post-ICH outcomes (SMD = 0.05–2.58, very low to low certainty), while progesterone had conflicting effects in ICH (SMDs = 0.15–1.16, very low to moderate certainty) and beneficial effects in SAH (SMD = 2.63, very low certainty). Estrogen and progesterone were mostly, though not unanimously, reported to be anti-inflammatory, anti-oxidative, anti-apoptotic, and to restore BBB integrity. Comparatively, studies reported testosterone to be both pro- and anti-apoptotic and inflammatory in almost equal measure, though research on the topic is generally lacking. Sex hormones were found to be mostly mediated by classical receptors, though estrogen was reported to have ER-independent actions as well. Sex and gonadal status moderated the effects of estrogen and progesterone across all outcomes, albeit to differing extents, with tentative evidence suggesting that delaying estrogen administration after gonadectomy diminishes estrogen’s protective effects on injury volume (i.e., the timing hypothesis). Qualitative analysis of our captured studies failed to suggest optimal dose parameters, with conflicting results across dose-response and target window studies. Importantly, our results should be interpreted with caution—scientifically and/or translationally rigorous studies were scarce, and some meta-analyses were based on very limited data (e.g., 3 studies). Overall, our meta-analysis suggests estrogen and progesterone may be promising cerebroprotectants following AIS, but highlights remaining avenues requiring further pre-clinical exploration.

**Fig. 10:**
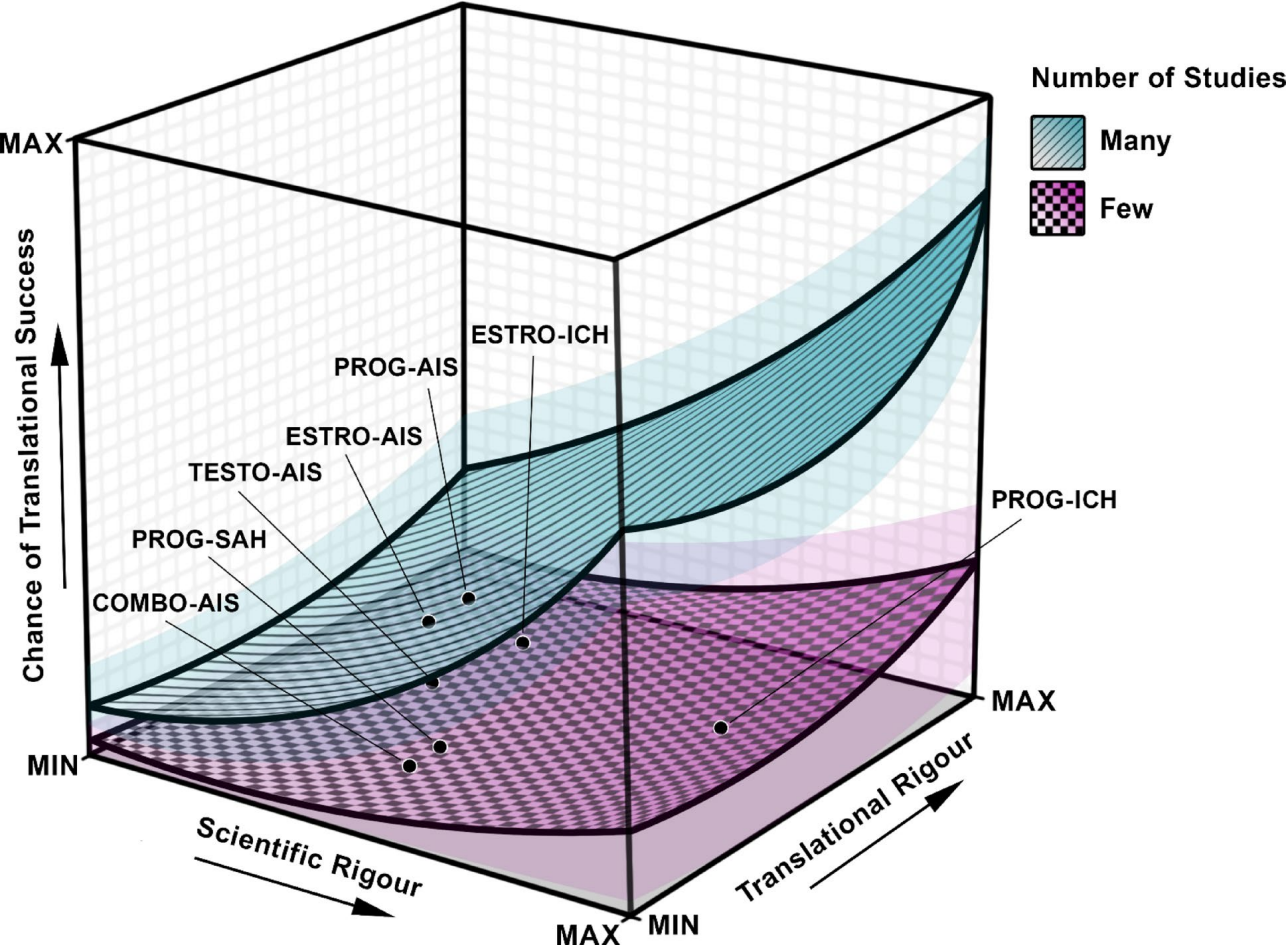
**The Three Dimensions of Translational Success**. The chance of translational success (y-axis) is likely influenced in a non-linear fashion by three factors: scientific rigor (x-axis), translational rigor (z-axis), and the number of studies (surface plots). Data points indicating the relative rigor of each literature base are provided. A literature base consisting of low-quality studies has a low probability of reflecting the true effect (beneficial, null, or harmful) that would be observed in clinical populations, regardless of the amount of data. However, a small number of high-quality studies also has a low probability of reflecting the true clinical effect, owing to the nature of inferential statistics. High scientific or translational rigor may independently increase translational success rate, but studies of high scientific and translational rigor likely have a much higher probability of accurately reflecting the true effect that would be observed in clinical populations. Overall, scientific rigor likely plays a more substantial role in translational success than translational rigor, though the latter is especially important in calculating clinically attainable effect size estimates

Sex and/or gonadal status consistently moderated the effects of estrogen and progesterone’s overall benefit, which could not be explored with testosterone. Estrogen invariably improved outcomes in OVX females, and progesterone in gonadally intact males. However, the biological importance of this moderation varied across endpoints. Sex and gonadal status explained anywhere from 0.02 to 99% of the variance between effect sizes, and groups were rarely significantly different from one another. Thus, sex and/or gonadal status are likely influential, though not independent moderators of estrogen and progesterone’s benefit, as would certainly be expected in patient populations (Fig. 10).

Gonadal depletion length was only a significant moderator of estrogen’s benefit on injury volume in AIS. In line with the timing hypothesis [1, 11], the longer the period of gonadal depletion, the less benefit was observed with estrogen administration. Importantly, most studies included in our meta-regression investigated short (≤ 3 week) delays, and thus the robustness of our reported relationship is not clear. Clinically, delays between menopause onset and HRT initiation varies widely [1]. Since studies commonly used OVX to model menopause, investigating longer gonadal depletion lengths will be valuable in increasing translational relevance to this population. Finally, it remains unclear whether gonadal depletion length moderates the effects of progesterone or testosterone, which could not be examined presently.

The effects of sex hormones are undoubtedly affected by additional factors beyond sex and gonadal status. Our exploratory analysis identified other potential variables affecting hormone efficacy, though the potential biological significance of some moderators (e.g., assessment test) is less clear than others (e.g., dosage, age). One notable moderator, age, is known to affect treatment efficacy [258], and currently differs substantially between the pre-clinical (mostly using young animals) and clinical literature (mostly consisting of elderly, post-menopausal women) [1, 11]. Likely, age and sex additionally interact, which may be of heightened relevance to patients prescribed HRT. Dosage and target window are undoubtedly key moderators as well. Exploratory meta-regressions suggest higher dosages of estrogen, lower dosages of progesterone, and later administration onset times to be associated with greater cytoprotection. However, this moderation was not observed consistently across all outcomes. Notably, we were unable to investigate interaction effects, which likely have a non-negligible effect and would have provided further insight to our findings. When exploring these factors qualitatively, we found that further research is needed to truly elucidate these effects. Only a handful of studies captured in our analysis directly investigated dose-response relationships or target windows of administration. Many dose-response studies investigated only two dosages, limiting the robustness of subsequent interpolation [259]. Further, many of our captured studies administered hormone in close proximity to stroke induction (i.e., pre-stroke or shortly post-stroke; see Tables S3.2, S3.3, and S3.4), which are difficult or impossible to achieve in clinical contexts [16, 254]. As pre-stroke and early post-stroke initiation is often associated with greater benefit with cytoprotective treatments, the prevalence of pre-and early post-stroke dosing regimens likely led to an overestimated effect size in our analysis compared to what may be achievable in patients [16, 254]. A lack of robust dose-response and target window research is an issue that is not unique to studies on hormones, and has been reported throughout the stroke field [16, 260]. Future animal research should explore these gaps in greater detail, as the evidence will be helpful in guiding the design (e.g., dosage parameters, patient selection) of early clinical work.

We failed to identify a harmful effect associated with the use of commercial slow release estrogen pellets in AIS [6] or a harmful effect of progesterone on mortality in AIS [5], both of which had been reported by prior meta-analyses. Potentially, these previous findings were spurious; these findings were based on 61 estrogen [6] and 19 progesterone [5] studies, compared to 128 and 53 studies in our analysis, respectively. Alternatively, publication bias may explain discrepant results. For example, there were slight indications of an ‘increase’ effect of estrogen on injury volume, suggesting the presence of recent unpublished null and/or negative data. Exclusion reporting in our progesterone data was similarly scarce, which could have skewed results. Alternatively, one may speculate that methodology has shifted over the years, such as more severe strokes perhaps being less commonly used in recent literature.

To maximize translational success of experimental treatments, a sizeable evidence base of sufficient scientifically (e.g., blinding, a priori sample size calculations) and translationally (e.g., aged animals and those with comorbidities, later administration onset times) rigorous studies is needed (Fig. 10). Despite considerable data, the certainty of the evidence throughout this meta-analysis is moderate, at best. Most of the evidence was of low or very low certainty, often due to low precision of estimates [22, 29], or low scientific or translational rigor. Typically, these qualities are hallmarks of inflated effect sizes and increased Type I error (false positive) rates [13, 16, 26, 255]. Notably, the most scientifically rigorous studies in our analysis tended to report null effects [55, 59, 112], though not unanimously [81, 113]. Surprisingly, domains of study quality were not independently associated with inflated effect sizes in our dataset, though the impact of blinding neared significance. Likely, a combination of quality domains (e.g., randomization, blinding, etc.), among other considerations (e.g., setting values for sample size calculations), contribute to inflated effect sizes, which are difficult to accurately assess in meta-analyses due to confounds of unclear reporting [27].

It is important to consider how much evidence of what quality is needed to maximize confidence in a treatment’s translational potential. One possibility is to only synthesize high quality evidence and pursue translation once these data suggest a high certainty of benefit. However, this approach introduces meaningful meta-bias, as determining an exact threshold for what constitutes a ‘high’ quality study is subjective. Alternatively, researchers have stratified therapies by the extent of testing as assessed by the STAIR criteria [17], though a similar issue emerges; no guidance exists to inform meta-analysts on how much data, and of what precision, are needed to consider a criteria fulfilled. For example, in our analysis, a maximum of four studies used animals with comorbidities in any one endpoint. These studies were not always of high scientific rigor, and thus still provide limited confidence in estrogen and progesterone’s translational success. Indeed, in our dataset, the most translationally rigorous studies were not typically the most scientifically rigorous, and vice versa. Thus, the most scientifically rigorous studies may have overestimated treatment efficacy due to low translational relevance (i.e., the use of young, healthy animals [258]), while the data with high translational rigor may have overestimated treatment efficacy due to low scientific quality (i.e., concerns surrounding researcher bias [13]). Due to these factors, the administration of estrogen and progesterone is unlikely to result in such substantial benefits in clinical contexts as has been observed in animal studies thus far.

The failure of the ProTECT [261] and SyNAPSe [262] Phase III clinical trials of progesterone in traumatic brain injury highlight important considerations in the design of future clinical studies, including a lack of attention to existing pre-clinical data [14]. Animal data can provide vital insight to clinicians in regards to efficacy and safety of experimental treatments [14, 17, 19]. Though the quality of pre-clinical studies varies widely, the pre-clinical evidence base is often manyfold larger than clinical evidence, and tests more diverse experimental conditions. Pre-clinical studies provide valuable data that can be used to inform trial design, such as target populations (e.g., sex and age), administration parameters (e.g., dosage), and endpoint selection (e.g., histological/mechanistic evaluation). To prevent similar pitfalls in the stroke field, collaborative efforts between pre-clinical and clinical researchers and meta-analysts are critical [19]. Efforts have been taken in recent decades to focus on high quality, translationally oriented, and confirmatory studies, aimed specifically at improving translational success rates. These efforts include the design and conduct of multi-laboratory pre-clinical RCTs, such as the Stroke Pre-Clinical Assessment Network [263].

Our meta-analysis has several limitations. First, only full-texts available in English were included, due to limitations of our research team. Second, given the lack of available raw data, we used parametric statistics to meta-analyze non-parametric data (e.g., ordinal neurological deficit scores), and converted non-parametric summary statistics (e.g., median) to mean and standard deviation for analysis. Though commonly done [38], this may have overestimated power and efficacy. Third, we did not include research group as a clustering variable within our analyses, primarily as this would have added an additional layer of complexity, likely with little additional discriminative value. However, it is probable that laboratories are more likely to report more consistent findings over time, owing to the continued use of similar models and standard operating procedures, or confirmation bias arising from previously conducted studies. Fourth, due to a considerable amount of published data, we did not include grey literature in our analyses. Although this may have provided greater perspective on the extent of unpublished null results, much of the grey literature captured in our searches were subsequently published and included in our analysis as full-texts.

## Conclusion

Overall, our meta-analysis updates the exogenous estrogen and progesterone literature, which has approximately doubled since the last conducted analyses [5, 6]. Our study is also the first to meta-analyze pre-clinical stroke studies on COMBO and testosterone in AIS, as well as any sex hormones in the context of ICH and SAH. We found estrogen and progesterone improved all outcomes following AIS, albeit with very low to moderate levels of certainty. Findings in ICH and SAH were conflicted, with greater benefit of progesterone (vs. estrogen) signalled in ICH, and generally low evidence certainty. Finally, testosterone effects on AIS were contradictory, although the evidence of its null effects ranged from very low to low certainty. Sex and gonadal status were moderators of estrogen and progesterone’s effects in AIS, suggesting future animal work should report and consider these variables in their investigations. Overall, our analysis suggests that estrogen and progesterone are cerebroprotective following AIS, though low study quality and limited scientific and translational rigor results in only moderate confidence in their translational potential, at best. Further high-quality investigation of critical research gaps (i.e., dose parameters) will be valuable in maximizing the translational potential of these hormones, and to potentially guide future clinical investigations.

**Table 1:** Sex hormone mechanisms of action in AIS

	AIS Mechanisms
Estrogen	• Inflammation o Anti-inflammatory [93, 96, 105, 138, 142, 149, 152, 160, 216, 217, 239, 246, 251] o No effect [50, 93, 162, 227] • Restored BBB integrity [53, 70, 163, 186, 217] • Cerebral Blood Flow o Increased CBF [111, 113, 146, 172, 184] o Cytoprotection dependent on CBF [111, 184] o Cytoprotection independent of CBF [113, 146, 172] o No effect [52, 111, 113, 117, 129, 135, 175, 176] • Oxidative stress o Anti-oxidative [96, 140, 148, 168, 186, 218, 245] o Cytoprotection independent of oxidative effects [243] o Pro-oxidative [92] • Cell death o Anti-apoptotic [44, 49, 67,103, 108, 109,114, 115, 116, 120, 122, 126, 128, 138, 145, 163, 241, 246, 249] o No effect on apoptosis [103] o Anti-autophagic [96] • Estrogen Receptor Involvement o ER-dependent [130, 138, 168, 172, 173, 245, 246] o ER-independent [154] o ERα-dependent [83, 125, 150, 167, 175, 251] o ERα-independent [164] o ERβ-dependent [125, 164] o ERβ-independent [129] o GPER-dependent [145, 246]
Progesterone	• Inflammation o Anti-inflammatory [66, 72, 85, 142, 169, 174, 190, 199, 201, 206, 211, 213] o Pro-inflammatory [66] • Restored BBB integrity [77, 151, 174, 190, 193, 199] • Cell death o Reduced cell death [209] o Anti-autophagic [155] o Anti-apoptotic [72, 155, 169, 174, 193] • Oxidative stress o Anti-oxidative [75, 169, 174, 205] o No effect [72] • Progesterone Receptor Involvement o PR-dependent [66, 211,212, 229] o PR-independent (allopregnanolone) [212]
Testosterone	• Inflammation o Anti-inflammatory [164, 222] o Pro-inflammatory [78] • Cell Death o Anti-apoptotic [165, 223] o Pro-apoptotic [78] • Overall Effect o Beneficial [62, 86, 90, 156, 222, 223, 238] o Harmful [58, 69, 78, 86, 165] • Androgen Receptor-dependent [58, 86, 156, 238]

Studies investigated a number of mechanisms aside from those meta-analyzed in our study, which are tabulated below

**Table 2:** Sex hormone mechanisms of action in ICH and SAH

	ICH Mechanisms	SAH Mechanisms
Estrogen	• Anti-inflammatory [48] • Restored BBB integrity [48, 54] • Anti-oxidative [48]	• Anti-inflammatory [43] • Restored BBB Integrity [43, 179] • Reduced cell death [43]
Progesterone	• Inflammation o Anti-inflammatory [198] o No effect [202,232] • Restored BBB integrity [198] • Reduced cell death [194] • Anti-oxidative [198]	• Anti-inflammatory [89,188] • Restored BBB integrity [188,192] • Reduced cell death [189,192] • Anti-oxidative [189]

Studies investigated a number of mechanisms aside from those meta-analyzed in our study, which are tabulated below

## Supplementary Information

Supplementary Material 1

Supplementary Material 2

Supplementary Material 3

Supplementary Material 4

Supplementary Material 5

## Data Availability

The datasets generated and analysed during the current study are available in the Open Science Framework repository (https://osf.io/kf7t2/overview).

## Abbreviations

AIS: Acute Ischemic Stroke
AR: Androgen Receptor
BBB: Blood-Brain Barrier
CAMARADES: Collaborative Approach to Meta-Analysis and Review of Animal Experimental Studies
CI: Confidence Interval
COMBO: Combination (Estrogen and Progesterone) Treatment
ER: Estrogen Receptor
GRADE: Grading of Recommendations, Assessment, Development, and Evaluation
HRT: Hormone Replacement Therapy
ICH: Intracerebral Hemorrhage
OVX: Ovariectomy
PI: Prediction Interval
PRISMA: Preferred Reporting Items for Systematic Reviews and Meta-Analyses
PR: Progesterone Receptor
PROSPERO: International Prospective Register of Systematic Reviews
SAH: Subarachnoid Hemorrhage
SMD: Standardized Mean Difference
STAIR: Stroke Therapy Academic Industry Roundtable
SYRCLE: Systematic Review Center for Laboratory Animal Experimentation
