# Supplementary material for: A comprehensive meta-analysis of exogenous estrogen, progesterone, and testosterone in animal models of ischemic and hemorrhagic stroke
Source: Biol Sex Differ. 2026 Jan 29;17:37. doi: 10.1186/s13293-026-00828-6 (PMC12924275; doi:10.1186/s13293-026-00828-6)
Supplement: Supplementary file 4 — Supplementary Material 4 [file 13293_2026_828_MOESM4_ESM.docx]

**A Comprehensive Meta-Analysis of Exogenous Estrogen, Progesterone, and Testosterone in Animal Models of Ischemic and Hemorrhagic Stroke**

Tiffany F. C. Kung, Angely Claire C. Suerte, Elmira Khiabani, Marin Parranto, Sara Gannon Arnott, Anna C. J. Kalisvaart, Shinichi Nakagawa, Ana C. Klahr, Frederick Colbourne

**Search Terms**

| Sex Hormone Search Terms | | |
| --- | --- | --- |
| A | B | C |
| estrogens OR estradiol | progesterone OR progestin OR progestins OR progesten OR progestogen OR pregnane OR norpregnane OR allopregnanolone | testosterone OR androgen OR nortestosterone or DHEA or dehydroepiandrosterone OR androsterone |
| Stroke Subtype Search Terms | | |
| 1 | 2 | 3 |
| intracerebral hemorrhage OR intracerebral haemorrhage OR intracranial hemorrhage OR intracranial haemorrhage OR ICH OR hemorrhagic stroke OR haemorrhagic stroke OR intraparenchymal hemorrhage OR intraparenchymal haemorrhage | focal ischemia OR focal ischaemia OR stroke OR ischemic stroke OR ischaemic stroke OR MCAO OR middle cerebral artery occlusion OR thromboembolic stroke OR atherothrombotic stroke OR occlusive stroke | subarachnoid hemorrhage OR subarachnoid haemorrhage OR SAH OR subarachnoid bleed OR SAB |
| Pre-Clinical Search Term | | |
| X | | |
| experimental OR pre-clinical OR rat OR rats OR mouse OR mice OR monkey OR monkeys OR animal OR animals OR animal model OR rodent OR rodents OR primate OR primates OR non-human OR murine | | |
|  |  |  |
| Searches were conducted on May 30, 2024 and May 1, 2025 on MEDLINE, EMBASE, Scopus and Web of Science. Alongside the pre-clinical search term, each sex hormone search term was combined with each stroke subtype search term for a total of 9 searches (i.e., A+1+X, A+2+X, A+3+X, B+1+X, B+2+X, B+3+X, C+1+X, C+2+X, C+3+X). | | |

**Modified GRADE-ing of Evidence Certainty**

Our analysis of evidence certainty followed GRADE guidelines with a few adaptations, discussed below [1]. Rather than initially grading evidence for randomization, we initially graded evidence based on a composite score, including: randomization, allocation concealment, blinding of outcome, selective outcome reporting, and other sources of bias (in our dataset, limited to industry sponsorship and selectively providing extractable data for significant outcomes only). Studies were given a score of 1 for ‘low risk of bias’, 0.5 for ‘some concerns’, and 0 for ‘high risk’. If the average of all study quality domains was ≥ 0.5, studies were initially rated as high certainty; if the average was < 0.5, studies were initially rated as low certainty.

Adherent to GRADE guidelines, studies were rated down for heterogeneity when significant in our meta-analytic model, and τ^2^ (between-study variance) ≥ 40%. Imprecision was graded using the minimally contextualized approach [2], using Hedge’s G = 0.26 (moderate effect [3]) as the minimally important difference. Briefly, studies were rated down once if 95% CIs crossed ± G = 0.26 on one side and rated down twice if 95% CIs crossed both -0.26 and 0.26. When studies did not cross ± 0.26, but indicated an unusually large effect, the optimal information size (OIS) was checked. The OIS was set as 241, the number of subjects needed to achieve 80% power and α = 0.05 to detect an effect size of G = 0.26 [3]. If the number of unique subjects (i.e., excluded repeated measures) exceeded 241, the study was not rated down. If the number of unique subjects was ≥ 50% of the OIS (i.e., n ≥ 120), the study was rated down once. If the number of unique subjects was ≥ 30% of the OIS (n ≥ 72), the study was rated down twice.

Studies were rated down for publication bias if there was significant evidence of publication bias, either via the small study effect or the decline effect. Studies were rated down for indirectness if < 50% of studies utilized translationally relevant animals.

**Detailed Statistical Analysis Methods**

This meta-analysis was conducted following the guides written by Yang, Nakagawa, and colleagues [4,5]. All analyses were performed in R studio, and the metafor, clubsandwich, orchard, ggplot2, robvis, patchwork, and metagear packages [6–13].

Where, necessary, median and IQR (formulas 11-12), median and range (formulas 9-10), means with 95% CIs (formulas 4-5) were converted to mean and standard deviation [14]. When values had a variance of 0, standard deviation was artificially set to 0.01. When variances were not provided, standard deviation was imputed using the metagear package [13]. Standardized mean differences were calculated rather than normalized mean differences, due to missing sham data. Standardized mean differences accounting for heteroscedastic population variances (i.e., `SMDH’ using the metafor package [7]) were estimated using Hedge’s G, are reported with 95% CI and 95% prediction intervals (PIs), as recommended [4,5]. Hormone dosages were converted to mg of hormone per kg of body weight; if animal weights were not given, weights were calculated according to average weights per animal age, according to strain and sex [15–23]. Permanent occlusions were manually set to 1000 minutes to allow for the analysis of occlusion length as a continuous variable. Hormone administration length was calculated from the time of first dose until the time of assessment (e.g., behavioural assessment or euthanasia for terminal outcomes). Continuous dosing methods and cortical grafts were assumed to last until euthanasia, and commercial pellets were assumed to last exactly as long as advertised. For all other dosing regimens whose administration lengths could not be determined, subcutaneous administrations were assumed to last for 96 hours, intramuscular for 72 hours, intraperitoneal and oral dosing for 24 hours, and intravenous dosing to last for 3 hours [24–28].

Exploratory meta-regressions were conducted in datasets with large amounts of data and sufficient studies per subgroup, to investigate the influence of potential moderators (e.g., age, time of first dose, hormone formulation, time of assessment, assessment test used, stroke severity, etc.). Significant moderators in the averaged model are reported in the main text, and model coefficients (B) are provided in the supplemental results (S3). Notably, these units are not standardized across models, and should not be compared to one another on an absolute scale [5,29]. Due to the large number of variables assessed, we were unable to further investigate the presence and influence of interaction effects within these datasets. Importantly, these results are obtained via data dredging and thus prone to Type I error [5]. Further *a priori* research on these moderators is necessary to confirm the findings of these exploratory analyses.

Univariate meta-analyses were performed on each endpoint. To account for dependent data arising from shared control groups, repeated measures study designs, and multiple treatment groups tested per study [4], hierarchical or cross-classified meta-analyses were used [4,5,30,31]. For all meta-analyses, a variance-covariance (VCV) matrix was applied to account for correlated data, using a ρ = 0.5 [4,5]. All hierarchical meta-analyses were assessed for robustness to model misspecification using robust variance estimation techniques [32]. All cross-classified random effects meta-analyses were assessed for robustness by setting a higher ρ of the VCV matrix (ρ = 0.8) [4,5].

As only one injury volume and edema assessment were extracted per group, three-level meta-analyses were conducted for these endpoints. For neurological deficits, four-level meta-analyses (effect sizes nested within ‘study’, nested within ‘assessment test’) were compared against three-level models using ANOVA, and the simplest, best-fitting model was used. For sensorimotor and cognitive outcomes, cross-classified random effects meta-analyses (effect sizes nested within ‘study’ and ‘assessment test’, with ‘study’ and ‘assessment test’ being crossed factors) were compared against three-level models using ANOVA, and the simplest, best-fitting model was used.

Heterogeneity was calculated using the I^2^ statistic, which was separated into between-study τ^2^ and within-study I^2^ where applicable. Meta-regressions were conducted where possible (i.e., where n ≥ 3 per subgroup) for the moderating effects of sex, gonadal status, and gonadal depletion length. Gonadal depletion length was calculated as the length of time from gonadal depletion (e.g., via ovariectomy) until hormone supplementation. A post-hoc meta-regression was conducted on administration route to follow up on findings from a previous meta-analysis [33]. Only administration methods used by at least 3 studies were included; thus, cortical grafts and intranasal administration were excluded from the meta-regression. Sensitivity analyses were conducted when obvious outlying estimates were obtained (e.g., Hedge’s G > 3000), which were typically observed due to low standard deviation values. Outliers were identified as studies with absolute studentized deleted residuals greater than ± 1.96, and their influence was investigated using sensitivity analyses [34].

Publication bias was investigated via the small study effect (i.e., whether smaller studies tend to report larger effect sizes) and the decline effect (i.e., whether effect sizes of a treatment tend to decay over time) [4,5]. Importantly, as almost all the studies included in our analysis used small group sizes, the small study effect should be interpreted carefully [5].

Mortality rates were meta-analyzed and reported as risk ratios. Only studies that provided determinable survival or mortality rates were meta-analyzed. Studies with difficult to determine survival rates (e.g., mortalities provided as percentages and total animal numbers unclear) were not meta-analyzed.

**References**

1. Hooijmans CR, de Vries RBM, Ritskes-Hoitinga M, Rovers MM, Leeflang MM, IntHout J, Wever KE, Hooft L, de Beer H, Kuijpers T, et al. Facilitating healthcare decisions by assessing the certainty in the evidence from preclinical animal studies. *PloS One*. 2018;13:e0187271.

2. Zeng L, Guyatt G. Updated GRADE guidance for imprecision rating using the minimally contextualized approach. 2022;

3. Brydges CR. Effect Size Guidelines, Sample Size Calculations, and Statistical Power in Gerontology. *Innov. Aging*. 2019;3:igz036.

4. Yang Y, Macleod M, Pan J, Lagisz M, Nakagawa S. Advanced methods and implementations for the meta-analyses of animal models: Current practices and future recommendations. *Neurosci. Biobehav. Rev.* 2023;146:105016.

5. Nakagawa S, Yang Y, Macartney EL, Spake R, Lagisz M. Quantitative evidence synthesis: a practical guide on meta-analysis, meta-regression, and publication bias tests for environmental sciences. *Environ. Evid.* 2023;12:8.

6. R Core Team. R: A language and environment for statistical computing [Internet]. 2024;Available from: https://www.R-project.org/

7. Viechtbauer W. Conducting Meta-Analyses in R with the metafor Package. *J. Stat. Softw.* [Internet]. 2010;36. Available from: http://www.jstatsoft.org/v36/i03/

8. Pustejovsky J. clubSandwich: Cluster-Robust (Sandwich) Variance Estimators with Small-Sample Corrections [Internet]. 2024;Available from: https://CRAN.R-project.org/package = clubSandwich

9. Nakagawa S, Lagisz M, O’Dea R, Pottier P, Rutkowska J, Senior A, Yang Y, Noble D. orchaRd 2.0: An R package for visualizing meta-analyses with orchard plots [Internet]. 2023 [cited 2024 Oct 25];Available from: https://ecoevorxiv.org/repository/view/4886/

10. Wickham H. ggplot2: Elegant Graphics for Data Analysis [Internet]. Springer-Verlag New York; 2016. Available from: https://ggplot2.tidyverse.org

11. McGuinness LA, Higgins JPT. Risk‐of‐bias VISualization (robvis): An R package and Shiny web app for visualizing risk‐of‐bias assessments. *Res. Synth. Methods*. 2021;12:55–61.

12. Lin Pedersen T. patchwork: The Composer of Plots [Internet]. 2024;Available from: https://CRAN.R-project.org/package = patchwork

13. Lajeunesse MJ. Facilitating systematic reviews, data extraction and meta‐analysis with the metagear package for r. *Methods Ecol. Evol.* 2016;7:323–330.

14. Chi K-Y, Li M-Y, Chen C, Kang E, Cochrane Taiwan. Ten circumstances and solutions for finding the sample mean and standard deviation for meta-analysis. *Syst. Rev.* 2023;12:62.

15. Konstantopoulos S. Fixed effects and variance components estimation in three-level meta-analysis: Three-level meta-analysis. *Res. Synth. Methods*. 2011;2:61–76.

16. Fernández-Castilla B, Maes M, Declercq L, Jamshidi L, Beretvas SN, Onghena P, Van Den Noortgate W. A demonstration and evaluation of the use of cross-classified random-effects models for meta-analysis. *Behav. Res. Methods*. 2019;51:1286–1304.

17. Pustejovsky JE, Tipton E. Meta-analysis with Robust Variance Estimation: Expanding the Range of Working Models. *Prev. Sci.* 2022;23:425–438.

18. Strom JO, Theodorsson A, Theodorsson E. Dose-Related Neuroprotective versus Neurodamaging Effects of Estrogens in Rat Cerebral Ischemia: A Systematic Analysis. *J. Cereb. Blood Flow Metab.* 2009;29:1359–1372.

19. Viechtbauer W, Cheung MW-L. Outlier and influence diagnostics for meta-analysis. *Res. Synth. Methods*. 2010;1:112–125.
