# Supplementary material for: A comprehensive meta-analysis of exogenous estrogen, progesterone, and testosterone in animal models of ischemic and hemorrhagic stroke
Source: Biol Sex Differ. 2026 Jan 29;17:37. doi: 10.1186/s13293-026-00828-6 (PMC12924275; doi:10.1186/s13293-026-00828-6)
Supplement: Supplementary file 5 — Supplementary Material 5 [file 13293_2026_828_MOESM5_ESM.docx]

**A Comprehensive Meta-Analysis of Exogenous Estrogen, Progesterone, and Testosterone in Animal Models of Ischemic and Hemorrhagic Stroke**

Tiffany F. C. Kung, Angely Claire C. Suerte, Elmira Khiabani, Marin Parranto, Sara Gannon Arnott, Anna C. J. Kalisvaart, Shinichi Nakagawa, Ana C. Klahr, Frederick Colbourne

**Captured Studies**

In total, 211 studies were included in our analysis [1–211]. Three studies were captured in our search, but were excluded as they only investigated combination treatments of hormone with another drug/regimen that was not tPA or rehabilitation [212–214].

**Non Meta-Analyzed Endpoints**

The results of findings that were not meta-analyzed due to insufficient data (i.e., < 3 studies), or endpoints that were extracted qualitatively (i.e., bleeding) are provided below. Endpoints that are not discussed in the main text or below were not investigated by studies captured within our search.

*COMBO on Sensorimotor Outcomes in AIS*

One study reported that a combination treatment of estrogen and progesterone significant improved sensorimotor outcomes after AIS [117].

*Estrogen on Injury Volume in ICH*

One study reported estrogen reduced injury volume in young intact males when administration began 2 weeks pre-stroke [9], while another reported no effect in OVX females when administration began 1 week post-stroke [97].

*Estrogen on Neurological Deficits in ICH*

Two studies found benefit of estrogen on neurological deficits at higher (0.2 and 0.3 mg/kg), but not lower (0.1 mg/kg) doses, when given 1h post-stroke [6,12]. One study assessed OVX females at 21d post-stroke [6], and the other assessed hyperglycemic males at 3d post-stroke [12].

*Estrogen on bleeding in ICH*

Two studies investigated the impact of estrogen on bleeding; one found estrogen to reduce hematoma volume at 12h [9], while the other found the hormone reduced hematoma expansion [12].

*Progesterone on Cognitive Outcomes in ICH*

One study reported progesterone to improve cognitive outcomes in gonadally intact males, but not gonadally intact females [160]. The same study found progesterone improved cognitive outcomes in OVX females at lower, but not higher doses (4 and 8 mg/kg vs. 16 mg/kg). Another study found no impact of progesterone on cognitive outcomes in gonadally intact males [46].

*Progesterone on bleeding in ICH*

One study reported progesterone administration to reduce hematoma volume [151], while two studies reported no effect [190,205].

*Estrogen on Injury Volume in SAH*

Two studies reported no effect of estrogen on SAH severity, one in gonadally intact males [1], and one in OVX females [4]. The second study reported that while estrogen did not affect the clot area, it did reduce secondary lesion volume.

*Estrogen on Edema and Neurological Deficits in SAH*

Two studies reported estrogen reduced edema and neurological deficits in gonadally intact males [1,137].

*Estrogen on Sensorimotor and Cognitive Outcomes in SAH*

One study reported that estrogen improved sensorimotor outcomes assessed via rotarod and beam walking scores and cognitive outcomes assessed via Morris Water Maze at later timepoints (i.e., 3d, 7d, and 22d respectively) [137].

*Progesterone on Injury Volume in SAH*

Two studies reported no effect of progesterone on injury volume in gonadally intact males [147,150].

*Progesterone on Edema in SAH*

Two studies reported progesterone reduced edema in gonadally intact males [146,150].

*Progesterone on Sensorimotor Outcomes in SAH*

One study reported progesterone given improved sensorimotor outcomes assessed via rotarod and grip strength at lower (8 mg/kg) but not higher (16 mg/kg) doses [47].

*Testosterone on Cognitive Outcomes in AIS*

Two studies reported that testosterone did not affect cognitive outcomes, assessed via the Y-Maze, object location memory test, and Morris Water Maze, and by the novel object recognition test, respectively [27,114].

*Testosterone on Edema and Sensorimotor Outcomes in ICH*

One study reported that that testosterone had no effect on edema, and worsened some sensorimotor outcomes (forelimb placing score), but not others (forelimb placing asymmetry) [60].

**Other Potential Moderators**

Other potential moderators of sex hormone effects were investigated in exploratory meta-regressions via model averaging. Notably, these results are prone to Type I error and should be interpreted with caution [215,216]; further *a priori* research is needed to confirm the direction and strength of these relationships.

*Estrogen in Ischemic Stroke*

Exploratory meta-regressions of the full dataset on injury volume identified no additional significant moderators, though hormone dosage neared significance (p = 0.052). Specifically, for each mg/kg in hormone dose, the efficacy of estrogen increased (model coefficient B = 0.004). In the dataset with outliers removed, dosage was a significant moderator (p = 0.04), in a similar direction (B = 0.004).

Exploratory meta-regressions were conducted on the full dataset on sensorimotor outcomes. Later times of administration onset (p < 0.001) and occlusion length (p < 0.001) were significant moderators such that for every hour delay between stroke onset and dosing initiation (B = 0.002) or minute increase of occlusion time (B = 0.001), estrogen efficacy increased. Conversely, use of the corner turn test (p = 0.008, B = -0.85) and forelimb placing test (p = 0.002, B = -1.19) were less likely to observe benefit of estrogen administration, while use of the rotarod test (p = 0.003, B = 1.75) was more likely to observe benefit. In the dataset with ouliers removed, only age was a significant moderator (p = 0.03), such that for every week increase in animal age, estrogen efficacy decreased (B = -0.02).

Model averaging was not done on edema or cognitive outcomes data due to lack of research. No significant moderators of estrogen’s effect on neurological deficit were identified via model averaging.

Lastly, we conducted subgroup analyses investigating whether administration of estrogen beginning either pre-dose or post-dose affected the hormone’s efficacy (Table S3.3). Indeed, pre-stroke dosing carries little translational relevance to the hormone’s efficacy as a post-stroke cytoprotectant, instead being more relevant to patients on hormone regimens at the time of stroke (e.g., stroke patients taking menopausal HRT). Overall, the time of dosing initiation, dichotomized to pre- or post-stroke, was a significant moderator of the overall effect of estrogen on injury volume, neurological deficits, and sensorimotor outcomes. However, none of these groups significantly differed from one another.

**Table S3.3. Estrogen Pre- vs Post-Stroke Dosing Onset Subgroup Analyses.** The results of subgroup analyses investigating whether the onset of estrogen dosing (dichotomized into dosing beginning pre-stroke or post-stroke) are provided below. Overall, a greater proportion of studies investigated pre-dose administrations than post-dose administrations.

| **Endpoint** | Overall Effect | Pre-Dose Effect | Post-Dose Effect | Did groups differ? |
| --- | --- | --- | --- | --- |
| Injury Volume | p < 0.001 | (k = 198, n = 97)  SMD = 0.864, 95% CI [0.708, 1.020], p < 0.001 | (k = 60, n = 35)  SMD = 0.878, 95% CI [0.601, 1.155], p < 0.001 | No (p = 0.928) |
| Injury Volume Sensitivity Analysis | p < 0.001 | (k = 170, n = 90)  SMD = 0.818, 95% CI [0.693, 0.944], p = 0.693 | (k = 56, n = 32)  SMD = 0.741, 95% CI [0.513, 0.968], p < 0.001 | No (p = 0.545) |
| Neurological Deficits | p < 0.001 | (k = 54, n = 27)  SMD = 0.837, 95% CI [0.607, 1.067], p < 0.001 | (k = 19, n = 11)  SMD = 0.904, 95% CI [0.461, 1.348], p < 0.001 | No (p = 0.789) |
| Sensorimotor Outcomes | p = 0.011 | (k = 113, n = 14)  SMD = 0.348, 95% CI [0.021, 0.675], p = 0.037 | (k = 34, n = 5)  SMD = 0.695, 95% CI [0.139, 1.251], p = 0.021 | No (p = 0.257) |
| Sensorimotor Outcomes Sensitivity Analysis | p = 0.010 | (k = 110, n = 14)  SMD = 0.302, 95% CI [0.097, 0.506], p = 0.004 | (k = 31, n = 5)  SMD = 0.241, 95% CI [-0.198, 0.681], p = 0.280 | No (p = 0.803) |

*Progesterone in Ischemic Stroke*

Data dredging of the injury volume data identified age (p = 0.01) and hormone dosage (p = 0.03) to be significant moderators. For every week increase in age (B = -0.01) and every mg/kg increase in hormone dose (B = 0.0007), progesterone efficacy decreased. The route of hormone administration neared significance, with subcutaneous administration more likely to observe benefit of progesterone (p = 0.07, B = 0.67).

Data dredging of the full sensorimotor outcome dataset identified length of hormone administration (p < 0.001), later times of administration onset (p = 0.001), and occlusion length (p = 0.04) to be significant moderators. For each hour increase in total progesterone administration length (B = 0.004), each hour delay in progesterone administration (B = 0.006), or each minute increase in occlusion duration (B = 0.0009), progesterone efficacy increased. Conversely, for every mg/kg increase in hormone dose (p = 0.003, B = -0.01) or every day delay in assessment time (p = 0.001, B = -0.04), progesterone efficacy decreased. Use of the grid walking test (p < 0.001, B = 1.02) or grip assessment (p = 0.02, B = 0.57) were more likely to observe benefit of progesterone.

Model averaging was not done on edema or cognitive outcomes data due to lack of research. No significant moderators of estrogen’s effect on neurological deficit were identified via model averaging.

We additionally investigated whether beginning progesterone administration pre- or post-stroke induction affected the efficacy of progesterone on outcomes (Table S3.4). Similar to estrogen, dosing initiation time, dichotomized as either beginning pre- or post-stroke, was a significant moderator of the overall effect of progesterone on injury volume, neurological deficits, and sensorimotor outcomes. However, these groups did not significantly differ in any endpoint except injury volume, where post-stroke initiation resulted in significantly greater benefit of progesterone than pre-stroke initiation. While this may suggest that initiating progesterone post-stroke onset may lead to greater benefit than pre-stroke dosing, differences between animal demographics and dosing concentrations suggest further research is needed.

**Table S3.4. Progesterone Pre- vs Post-Stroke Dosing Onset Subgroup Analyses.** The results of subgroup analyses investigating whether the onset of progesterone dosing (dichotomized into dosing beginning pre-stroke or post-stroke) are provided below. Overall, a greater proportion of studies investigated post-dose administrations than pre-dose administrations.

| **Endpoint** | Overall Effect | Pre-Dose Effect | Post-Dose Effect | Did groups differ? |
| --- | --- | --- | --- | --- |
| Injury Volume | p < 0.001 | (k = 18, n = 11)  SMD = 0.578, 95% CI [0.204, 0.951], p = 0.003 | (k = 80, n = 41)  SMD = 1.098, 95% CI [0.878, 1.318], p < 0.001 | Yes (p = 0.011). |
| Neurological Deficits | p < 0.001 | (k = 11, n = 4)  SMD = 0.431, 95% CI [0.021, 0.840], p = 0.039 | (k = 87, n = 17)  SMD = 0.715, 95% CI [0.455, 0.976], p < 0.001 | No (p = 0.148) |
| Sensorimotor Outcomes | p = 0.001 | (k = 6, n = 2)  SMD = 0.095, 95% CI [-0.709, 0.899], p = 0.816 | (k = 381, n = 28)  SMD = 0.550, 95% CI [0.225, 0.876], p < 0.001 | No (p = 0.250) |
| Sensorimotor Outcomes Sensitivity Analysis | p < 0.001 | (k = 6, n = 2)  SMD = 0.451, 95% CI [-0.147, 1.049], p = 0.139 | (k = 367, n = 27)  SMD = 0.677, 95% CI [0.493, 0.861], p < 0.001 | No (p = 0.465) |

The results of these exploratory meta-regressions and subgroup analyses should be interpreted with caution. Few of these findings are consistent across multiple endpoints, suggesting further research is needed to understand the true moderating effects of these variables. Overall, our exploratory meta-regressions and unplanned subgroup analyses are at higher risk for Type I error associated with family-wise error. Finally, it is likely that many of these variables are interrelated, and affect each other through complicated interaction effects and relationships, which we were unable to investigate with our current analysis.

*
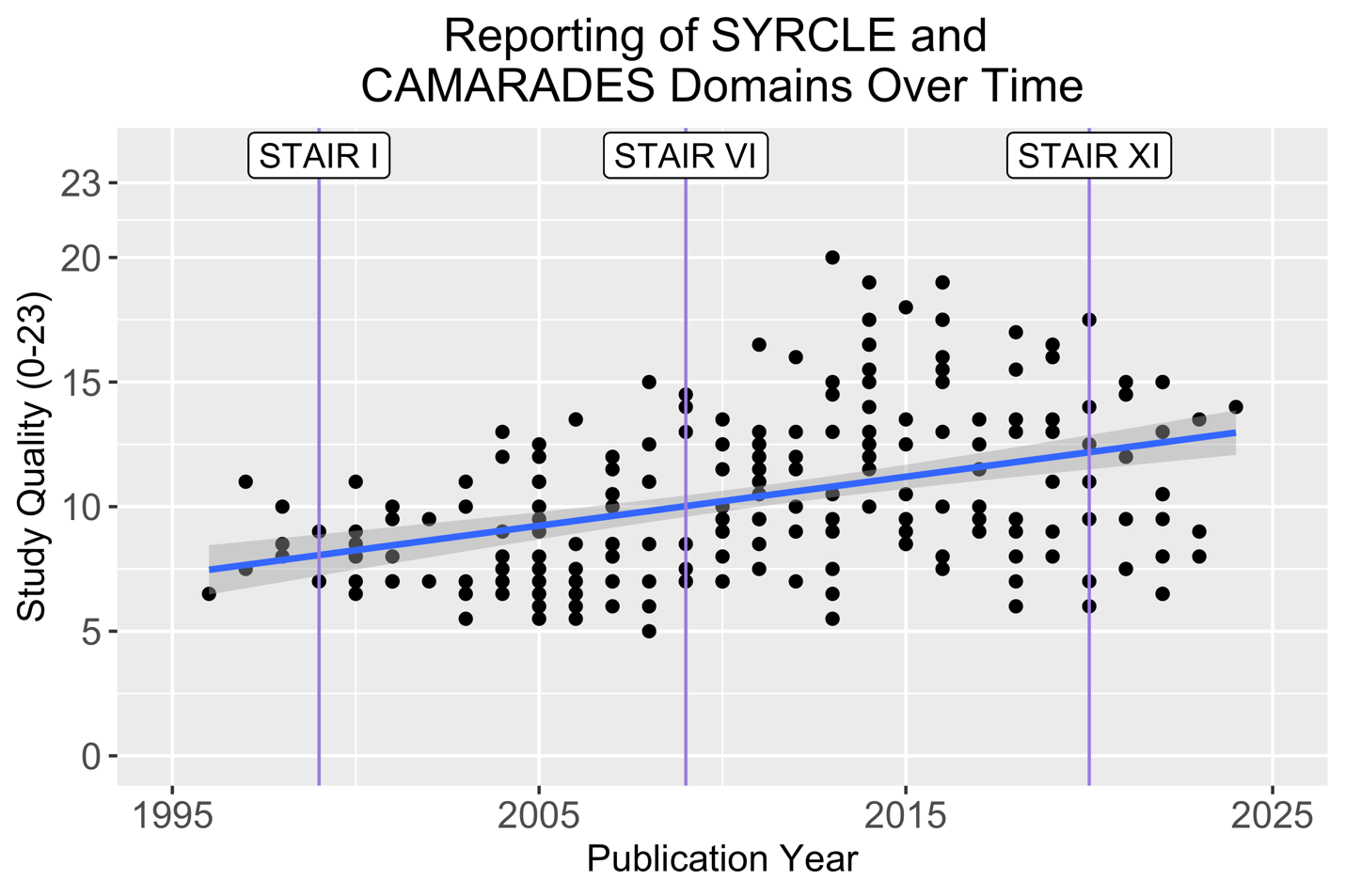
*

**Figure S3.1. Reporting of SYRCLE and CAMARADES Domains Over Time.** Overall, the reporting of the domains assessed by SYRCLE and CAMARADES improved over time (R^2^ = 0.16, p < 0.001). Specifically, while recent studies have demonstrated higher domain reporting than earlier studies, the prevalence of studies with low domain reporting remains relatively similar. The publication of pre-clinical STAIR guidelines have been indicated, namely the STAIR I [217], STAIR VI [218], and STAIR XI [219] reports. Overall, studies published in 2010 or later (following the publication of the widely cited STAIR VI; mean = 11.6) show significantly higher reporting of SYRCLE and CAMARADES domains than studies published before (mean = 8.7; p < 0.001). Importantly, these results should be interpreted with caution, as higher scores on this scale do not necessarily indicate higher quality; indeed, the quantification of scales such as SYRCLE and CAMARADES are discouraged [220].

**References**

1. Hu Q, Du Q, Yu W, Dong X. 2-Methoxyestradiol Alleviates Neuroinflammation and Brain Edema in Early Brain Injury After Subarachnoid Hemorrhage in Rats. Front Cell Neurosci [Internet]. 2022;16. https://doi.org/10.3389/fncel.2022.869546

2. Choi YC, Lee JH, Hong KW, Lee KS. 17 Beta-estradiol prevents focal cerebral ischemic damages via activation of Akt and CREB in association with reduced PTEN phosphorylation in rats. Fundam Clin Pharmacol. England; 2004;18:547–57. https://doi.org/10.1111/j.1472-8206.2004.00284.x

3. Cheng Y, Su Q, Shao B, Cheng J, Wang H, Wang L, et al. 17 β -Estradiol attenuates poststroke depression and increases neurogenesis in female ovariectomized rats. BioMed Res Int. United States; 2013;2013:392434. https://doi.org/10.1155/2013/392434

4. Yang SH, He Z, Wu SS, He YJ, Cutright J, Millard WJ, et al. 17-beta estradiol can reduce secondary ischemic damage and mortality of subarachnoid hemorrhage. J Cereb Blood Flow Metab. United States; 2001;21:174–81. https://doi.org/10.1097/00004647-200102000-00009

5. Söderström I, Strand M, Ingridsson AC, Nasic S, Olsson T. 17beta-estradiol and enriched environment accelerate cognitive recovery after focal brain ischemia. Eur J Neurosci. France; 2009;29:1215–24. https://doi.org/10.1111/j.1460-9568.2009.06662.x

6. Xiao H., Liu J., He J., Lan Z., Deng M., Hu Z. 17beta-Estradiol Attenuates Intracerebral Hemorrhage-Induced Blood-Brain Barrier Injury and Oxidative Stress Through SRC3-Mediated PI3K/Akt Signaling Pathway in a Mouse Model. ASN Neuro [Internet]. United Kingdom: SAGE Publications Inc.; 2021;13. https://doi.org/10.1177/17590914211038443

7. Fan T, Yang S-H, Johnson E, Osteen B, Hayes R, Day AL, et al. 17beta-Estradiol extends ischemic thresholds and exerts neuroprotective effects in cerebral subcortex against transient focal cerebral ischemia in rats. Brain Res. Netherlands; 2003;993:10–7. https://doi.org/10.1016/j.brainres.2003.07.006

8. Yong Y, Xie HJ, Zhang YF, Yang QD, Liao DF, Yang HL, et al. 17beta-estradiol potentiates ischemia-reperfusion injury in diabetic ovariectomized female rats. Brain Res. Netherlands; 2005;1054:192–9. https://doi.org/10.1016/j.brainres.2005.05.069

9. Auriat A, Plahta WC, McGie SC, Yan R, Colbourne F. 17beta-Estradiol pretreatment reduces bleeding and brain injury after intracerebral hemorrhagic stroke in male rats. J Cereb Blood Flow Metab. United States; 2005;25:247–56. https://doi.org/10.1038/sj.jcbfm.9600026

10. Rusa R, Alkayed NJ, Crain BJ, Traystman RJ, Kimes AS, London ED, et al. 17beta-estradiol reduces stroke injury in estrogen-deficient female animals. Stroke. United States; 1999;30:1665–70. https://doi.org/10.1161/01.str.30.8.1665

11. Li M, Zhang Z, Sun W, Koehler RC, Huang J. 17β-estradiol attenuates breakdown of blood-brain barrier and hemorrhagic transformation induced by tissue plasminogen activator in cerebral ischemia. Neurobiol Dis. United States; 2011;44:277–83. https://doi.org/10.1016/j.nbd.2011.07.004

12. Zheng Y, Hu Q, Manaenko A, Zhang Y, Peng Y, Xu L, et al. 17β-Estradiol attenuates hematoma expansion through estrogen receptor α/silent information regulator 1/nuclear factor-kappa b pathway in hyperglycemic intracerebral hemorrhage mice. Stroke. United States; 2015;46:485–91. https://doi.org/10.1161/STROKEAHA.114.006372

13. Farr TD, Carswell HVO, Gallagher L, Condon B, Fagan AJ, Mullin J, et al. 17β-Estradiol treatment following permanent focal ischemia does not influence recovery of sensorimotor function. Neurobiol Dis. 2006;23:552–62. https://doi.org/10.1016/j.nbd.2006.04.009

14. Connell BJ, Saleh TM. A novel rodent model of reperfusion injury following occlusion of the middle cerebral artery. J Neurosci Methods. Netherlands; 2010;190:28–33. https://doi.org/10.1016/j.jneumeth.2010.04.021

15. Ritz M-F, Schmidt P, Mendelowitsch A. Acute effects of 17beta-estradiol on the extracellular concentration of excitatory amino acids and energy metabolites during transient cerebral ischemia in male rats. Brain Res. Netherlands; 2004;1022:157–63. https://doi.org/10.1016/j.brainres.2004.07.004

16. Cheng J, Hu W, Toung TJ, Zhang Z, Parker SM, Roselli CE, et al. Age-dependent effects of testosterone in experimental stroke. J Cereb Blood Flow Metab. United States; 2009;29:486–94. https://doi.org/10.1038/jcbfm.2008.138

17. Spratt NJ, Tomkins AJ, Pepperall D, McLeod DD, Calford MB. Allopregnanolone and its precursor progesterone do not reduce injury after experimental stroke in hypertensive rats - role of postoperative temperature regulation? PLoS One. United States; 2014;9:e107752. https://doi.org/10.1371/journal.pone.0107752

18. Sayeed I, Guo Q, Hoffman SW, Stein DG. Allopregnanolone, a progesterone metabolite, is more effective than progesterone in reducing cortical infarct volume after transient middle cerebral artery occlusion. Ann Emerg Med. United States; 2006;47:381–9. https://doi.org/10.1016/j.annemergmed.2005.12.011

19. Shimizu T., MacEy T.A., Quillinan N., Klawitter J., Perraud A.-L.L., Traystman R.J., et al. Androgen and PARP-1 regulation of TRPM2 channels after ischemic injury. J Cereb Blood Flow Metab. United States: Nature Publishing Group (345 Park Avenue South, New York NY 10010-1707, United States); 2013;33:1549–55. https://doi.org/10.1038/jcbfm.2013.105

20. Ayala P., Uchida M., Akiyoshi K., Cheng J., Hashimoto J., Jia T., et al. Androgen Receptor Overexpression Is Neuroprotective in Experimental Stroke. Transl Stroke Res. United States: Springer US; 2011;2:346–57. https://doi.org/10.1007/s12975-011-0079-z

21. Wang J., Zhou Y., He L. Appropriate supplementation of testosterone alleviates post-stroke damage via decreasing inflammation and oxidative stress in aged male C57BL/6 mice. Eur J Inflamm [Internet]. United Kingdom: SAGE Publications Inc.; 2022;20. https://doi.org/10.1177/1721727X221116739

22. McCullough LD, Blizzard K, Simpson ER, Oz OK, Hurn PD. Aromatase cytochrome P450 and extragonadal estrogen play a role in ischemic neuroprotection. J Neurosci. United States; 2003;23:8701–5. https://doi.org/10.1523/JNEUROSCI.23-25-08701.2003

23. Liu B-Y, Song X-L, Yi J, Chen X-M, Yu Y, Liu H, et al. Buyang Huanwu Decoction reduces infarct volume and enhances estradiol and estradiol receptor concentration in ovariectomized rats after middle cerebral artery occlusion. Chin J Integr Med. 2014;20:782–6. https://doi.org/10.1007/s11655-014-1823-5

24. Zhu X, Fréchou M, Schumacher M, Guennoun R. Cerebroprotection by progesterone following ischemic stroke: Multiple effects and role of the neural progesterone receptors. J Steroid Biochem Mol Biol. England; 2019;185:90–102. https://doi.org/10.1016/j.jsbmb.2018.07.014

25. Burguete M.C., Jover-Mengual T., Castello-Ruiz M., Lopez-Morales M.A., Centeno J.M., Aliena-Valero A., et al. Cerebroprotective Effect of 17beta-Estradiol Replacement Therapy in Ovariectomy-Induced Post-Menopausal Rats Subjected to Ischemic Stroke: Role of MAPK/ERK1/2 Pathway and PI3K-Independent Akt Activation. Int J Mol Sci. Switzerland: Multidisciplinary Digital Publishing Institute (MDPI); 2023;24:14303. https://doi.org/10.3390/ijms241814303

26. Li X, Blizzard KK, Zeng Z, DeVries AC, Hurn PD, McCullough LD. Chronic behavioral testing after focal ischemia in the mouse: functional recovery and the effects of gender. Exp Neurol. 2004;187:94–104. https://doi.org/10.1016/j.expneurol.2004.01.004

27. Smith C, Contreras-Garza J, Cunningham RL, Wong JM, Vann PH, Metzger D, et al. Chronic Testosterone Deprivation Sensitizes the Middle-Aged Rat Brain to Damaging Effects of Testosterone Replacement. Neuroendocrinology. Switzerland; 2020;110:914–28. https://doi.org/10.1159/000504445

28. Liu R, Liu Q, He S, Simpkins JW, Yang SH. Combination therapy of 17beta-estradiol and recombinant tissue plasminogen activator for experimental ischemic stroke. J Pharmacol Exp Ther. United States; 2010;332:1006–12. https://doi.org/10.1124/jpet.109.160937

29. Wang J, Feng X, Du Y, Wang L, Zhang S. Combination treatment with progesterone and rehabilitation training further promotes behavioral recovery after acute ischemic stroke in mice. Restor Neurol Neurosci. Netherlands; 2013;31:487–99. https://doi.org/10.3233/RNN-130312

30. Atif F, Yousuf S, Sayeed I, Ishrat T, Hua F, Stein DG. Combination treatment with progesterone and vitamin D hormone is more effective than monotherapy in ischemic stroke: the role of BDNF/TrkB/Erk1/2 signaling in neuroprotection. Neuropharmacology. England; 2013;67:78–87. https://doi.org/10.1016/j.neuropharm.2012.10.004

31. Hoffmann S, Beyer C, Zendedel A. Comparative analysis of gonadal steroid-mediated neuroprotection after transient focal ischemia in rats: route of application and substrate composition. J Mol Neurosci MN. United States; 2015;56:12–6. https://doi.org/10.1007/s12031-014-0462-9

32. Shin JA, Choi JH, Choi Y-H, Park E-M. Conserved aquaporin 4 levels associated with reduction of brain edema are mediated by estrogen in the ischemic brain after experimental stroke. Biochim Biophys Acta. Netherlands; 2011;1812:1154–63. https://doi.org/10.1016/j.bbadis.2011.05.004

33. Lee JY, Castelli V, Kumar N, Sitruk-Ware R, Borlongan CV. Contraceptive drug, Nestorone, enhances stem cell-mediated remodeling of the stroke brain by dampening inflammation and rescuing mitochondria. Free Radic Biol Med. United States; 2022;183:138–45. https://doi.org/10.1016/j.freeradbiomed.2022.03.020

34. El Amki M, Binder N, Steffen R, Schneider H, Luft AR, Weller M, et al. Contraceptive drugs mitigate experimental stroke-induced brain injury. Cardiovasc Res. England; 2019;115:637–46. https://doi.org/10.1093/cvr/cvy248

35. Yousuf S, Sayeed I, Atif F, Tang H, Wang J, Stein DG. Delayed progesterone treatment reduces brain infarction and improves functional outcomes after ischemic stroke: a time-window study in middle-aged rats. J Cereb Blood Flow Metab. United States; 2014;34:297–306. https://doi.org/10.1038/jcbfm.2013.198

36. Cheng J, Alkayed NJ, Hurn PD. Deleterious effects of dihydrotestosterone on cerebral ischemic injury. J Cereb Blood Flow Metab. United States; 2007;27:1553–62. https://doi.org/10.1038/sj.jcbfm.9600457

37. Bingham D, Macrae IM, Carswell HV. Detrimental effects of 17beta-oestradiol after permanent middle cerebral artery occlusion. J Cereb Blood Flow Metab. United States; 2005;25:414–20. https://doi.org/10.1038/sj.jcbfm.9600031

38. Oppong-Gyebi A, Metzger D, Vann PH, Yockey RA, Sumien N, Schreihofer DA. Dietary genistein and 17β-estradiol implants differentially influence locomotor and cognitive functions following transient focal ischemia in middle-aged ovariectomized rats at different lengths of estrogen deprivation. Horm Behav. United States; 2022;144:105201. https://doi.org/10.1016/j.yhbeh.2022.105201

39. Strom JO, Theodorsson E, Holm L, Theodorsson A. Different methods for administering 17β-estradiol to ovariectomized rats result in opposite effects on ischemic brain damage. BMC Neurosci [Internet]. 2010;11. https://doi.org/10.1186/1471-2202-11-39

40. Carswell HV, Bingham D, Wallace K, Nilsen M, Graham DI, Dominiczak AF, et al. Differential Effects of 17β-Estradiol Upon Stroke Damage in Stroke Prone and Normotensive Rats. J Cereb Blood Flow Metab. 2004;24:298–304. https://doi.org/10.1097/01.WCB.0000112322.75217.FD

41. Dubal DB, Rau SW, Shughrue PJ, Zhu H, Yu J, Cashion AB, et al. Differential modulation of estrogen receptors (ERs) in ischemic brain injury: a role for ERalpha in estradiol-mediated protection against delayed cell death. Endocrinology. United States; 2006;147:3076–84. https://doi.org/10.1210/en.2005-1177

42. Liu R, Wang X, Liu Q, Yang S-H, Simpkins JW. Dose dependence and therapeutic window for the neuroprotective effects of 17beta-estradiol when administered after cerebral ischemia. Neurosci Lett. Ireland; 2007;415:237–41. https://doi.org/10.1016/j.neulet.2007.01.074

43. Fréchou M, Zhu X, Liere P, Pianos A, Schumacher M, Mattern C, et al. Dose-dependent and long-term cerebroprotective effects of intranasal delivery of progesterone after ischemic stroke in male mice. Neuropharmacology. England; 2020;170:108038. https://doi.org/10.1016/j.neuropharm.2020.108038

44. Uchida M, Palmateer JM, Herson PS, DeVries AC, Cheng J, Hurn PD. Dose-dependent effects of androgens on outcome after focal cerebral ischemia in adult male mice. J Cereb Blood Flow Metab. United States; 2009;29:1454–62. https://doi.org/10.1038/jcbfm.2009.60

45. Goodrow GJ, Vitullo L, Cipolla MJ. Effect of estrogen therapy on cerebral arteries during stroke in female rats. Menopause. United States; 2005;12:99–109. https://doi.org/10.1097/00042192-200512010-00017

46. Lyden P, Shin C, Jackson-Friedman C, Hassid S, Chong A, Macdonald RL. Effect of ganaxolone in a rodent model of cerebral hematoma. Stroke. United States; 2000;31:169–75. https://doi.org/10.1161/01.str.31.1.169

47. Turan N, Miller BA, Huie JR, Heider RA, Wang J, Wali B, et al. Effect of Progesterone on Cerebral Vasospasm and Neurobehavioral Outcomes in a Rodent Model of Subarachnoid Hemorrhage. World Neurosurg. 2018;110:e150–9. https://doi.org/10.1016/j.wneu.2017.10.118

48. Pan Y, Zhang H, Acharya AB, Patrick PH, Oliver D, Morley JE. Effect of testosterone on functional recovery in a castrate male rat stroke model. Brain Res. Netherlands; 2005;1043:195–204. https://doi.org/10.1016/j.brainres.2005.02.078

49. Shi J, Zhang YQ, Simpkins JW. Effects of 17beta-estradiol on glucose transporter 1 expression and endothelial cell survival following focal ischemia in the rats. Exp Brain Res. Germany; 1997;117:200–6. https://doi.org/10.1007/s002210050216

50. Gordon KB, Macrae IM, Carswell HVO. Effects of 17β-oestradiol on cerebral ischaemic damage and lipid peroxidation. Brain Res. 2005;1036:155–62. https://doi.org/10.1016/j.brainres.2004.12.052

51. Liu F, Benashski SE, Xu Y, Siegel M, McCullough LD. Effects of chronic and acute oestrogen replacement therapy in aged animals after experimental stroke. J Neuroendocrinol. United States; 2012;24:319–30. https://doi.org/10.1111/j.1365-2826.2011.02248.x

52. Toung TJ, Chen TY, Littleton-Kearney MT, Hurn PD, Murphy SJ. Effects of combined estrogen and progesterone on brain infarction in reproductively senescent female rats. J Cereb Blood Flow Metab. United States; 2004;24:1160–6. https://doi.org/10.1097/01.WCB.0000135594.13576.D2

53. Littleton-Kearney MT, Klaus JA, Hurn PD. Effects of combined oral conjugated estrogens and medroxyprogesterone acetate on brain infarction size after experimental stroke in rat. J Cereb Blood Flow Metab. United States; 2005;25:421–6. https://doi.org/10.1038/sj.jcbfm.9600052

54. Li L, Chen J, Sun S, Zhao J, Dong X, Wang J. Effects of Estradiol on Autophagy and Nrf-2/ARE Signals after Cerebral Ischemia. Cell Physiol Biochem Int J Exp Cell Physiol Biochem Pharmacol. Germany; 2017;41:2027–36. https://doi.org/10.1159/000475433

55. Zhang YQ, Shi J, Rajakumar G, Day AL, Simpkins JW. Effects of gender and estradiol treatment on focal brain ischemia. Brain Res. Netherlands; 1998;784:321–4. https://doi.org/10.1016/s0006-8993(97)00502-7

56. Ingberg E, Theodorsson E, Theodorsson A, Ström JO. Effects of high and low 17β-estradiol doses on focal cerebral ischemia in rats. Sci Rep. England; 2016;6:20228. https://doi.org/10.1038/srep20228

57. Strom JO, Ingberg E, Theodorsson E, Theodorsson A. Effects of high and low 17β-estradiol doses on focal cerebral ischemia: negative results. Sci Rep. England; 2013;3:3111. https://doi.org/10.1038/srep03111

58. Wang Ming, Lu Ya-ping, Zhu Guo-ping, Zhang Xiao-pan, Han Ying, Yu Zhong-bing. Effects of oestrogen on ischemia-induced neurogenesis in the dentate gyrus of rats. Zool Res. 2007;28:88–94.

59. Ishrat T, Sayeed I, Atif F, Stein DG. Effects of progesterone administration on infarct volume and functional deficits following permanent focal cerebral ischemia in rats. Brain Res. Netherlands; 2009;1257:94–101. https://doi.org/10.1016/j.brainres.2008.12.048

60. Chen Z, Xi G, Mao Y, Keep RF, Hua Y. Effects of progesterone and testosterone on ICH-induced brain injury in rats. Acta Neurochir Suppl. Austria; 2011;111:289–93. https://doi.org/10.1007/978-3-7091-0693-8_48

61. Ma Y, Niu E, Xie F, Liu M, Sun M, Peng Y, et al. Electroacupuncture reactivates estrogen receptors to restore the neuroprotective effect of estrogen against cerebral ischemic stroke in long-term ovariectomized rats. Brain Behav. United States; 2021;11:e2316. https://doi.org/10.1002/brb3.2316

62. Westberry JM, Prewitt AK, Wilson ME. Epigenetic regulation of the estrogen receptor alpha promoter in the cerebral cortex following ischemia in male and female rats. Neuroscience. United States; 2008;152:982–9. https://doi.org/10.1016/j.neuroscience.2008.01.048

63. Zhang B, Subramanian S, Dziennis S, Jia J, Uchida M, Akiyoshi K, et al. Estradiol and G1 reduce infarct size and improve immunosuppression after experimental stroke. J Immunol Baltim Md 1950. United States; 2010;184:4087–94. https://doi.org/10.4049/jimmunol.0902339

64. Perez-Alvarez MJ, Mateos L, Alonso A, Wandosell F. Estradiol and Progesterone Administration After pMCAO Stimulates the Neurological Recovery and Reduces the Detrimental Effect of Ischemia Mainly in Hippocampus. Mol Neurobiol. United States; 2015;52:1690–703. https://doi.org/10.1007/s12035-014-8963-7

65. Wang L, Kitano H, Hurn PD, Murphy SJ. Estradiol attenuates neuroprotective benefits of isoflurane preconditioning in ischemic mouse brain. J Cereb Blood Flow Metab. United States; 2008;28:1824–34. https://doi.org/10.1038/jcbfm.2008.70

66. Rau SW, Dubal DB, Böttner M, Gerhold LM, Wise PM. Estradiol attenuates programmed cell death after stroke-like injury. J Neurosci Off J Soc Neurosci. United States; 2003;23:11420–6. https://doi.org/10.1523/JNEUROSCI.23-36-11420.2003

67. Koh PO, Cho JH, Won CK, Lee HJ, Sung JH, Kim MO. Estradiol attenuates the focal cerebral ischemic injury through mTOR/p70S6 kinase signaling pathway. Neurosci Lett. Ireland; 2008;436:62–6. https://doi.org/10.1016/j.neulet.2008.02.061

68. Ardelt AA, Anjum N, Rajneesh KF, Kulesza P, Koehler RC. Estradiol augments peri-infarct cerebral vascular density in experimental stroke. Exp Neurol. United States; 2007;206:95–100. https://doi.org/10.1016/j.expneurol.2007.04.002

69. Yang SH, Shi J, Day AL, Simpkins JW. Estradiol exerts neuroprotective effects when administered after ischemic insult. Stroke. United States; 2000;31:745–9; discussion 749. https://doi.org/10.1161/01.str.31.3.745

70. Theodorsson A, Theodorsson E. Estradiol increases brain lesions in the cortex and lateral striatum after transient occlusion of the middle cerebral artery in rats: no effect of ischemia on galanin in the stroke area but decreased levels in the hippocampus. Peptides. United States; 2005;26:2257–64. https://doi.org/10.1016/j.peptides.2005.04.013

71. Ardelt AA, Carpenter RS, Lobo MR, Zeng H, Solanki RB, Zhang A, et al. Estradiol modulates post-ischemic cerebral vascular remodeling and improves long-term functional outcome in a rat model of stroke. Brain Res. Netherlands; 2012;1461:76–86. https://doi.org/10.1016/j.brainres.2012.04.024

72. Koh PO. Estradiol prevents the injury-induced decrease of 90 ribosomal S6 kinase (p90RSK) and Bad phosphorylation. Neurosci Lett. Ireland; 2007;412:68–72. https://doi.org/10.1016/j.neulet.2006.10.060

73. Won CK, Ha SJ, Noh HS, Kang SS, Cho GJ, Choi WS, et al. Estradiol prevents the injury-induced decrease of Akt activation and Bad phosphorylation. Neurosci Lett. Ireland; 2005;387:115–9. https://doi.org/10.1016/j.neulet.2005.07.021

74. Koh PO, Won CK, Cho JH. Estradiol prevents the injury-induced decrease of Akt/glycogen synthase kinase 3beta phosphorylation. Neurosci Lett. Ireland; 2006;404:303–8. https://doi.org/10.1016/j.neulet.2006.06.020

75. Dubal DB, Kashon ML, Pettigrew LC, Ren JM, Finklestein SP, Rau SW, et al. Estradiol protects against ischemic injury. J Cereb Blood Flow Metab. United States; 1998;18:1253–8. https://doi.org/10.1097/00004647-199811000-00012

76. O’Donnell ME, Lam TI, Tran LQ, Foroutan S, Anderson SE. Estradiol reduces activity of the blood-brain barrier Na-K-Cl cotransporter and decreases edema formation in permanent middle cerebral artery occlusion. J Cereb Blood Flow Metab. United States; 2006;26:1234–49. https://doi.org/10.1038/sj.jcbfm.9600278

77. Jiang H, Xiao L, Jin K, Shao B. Estrogen administration attenuates post-stroke depression by enhancing CREB/BDNF/TrkB signaling in the rat hippocampus. Exp Ther Med. 2021;21. https://doi.org/10.3892/etm.2021.9850

78. Alkayed NJ, Goto S, Sugo N, Joh HD, Klaus J, Crain BJ, et al. Estrogen and Bcl-2: gene induction and effect of transgene in experimental stroke. J Neurosci Off J Soc Neurosci. United States; 2001;21:7543–50. https://doi.org/10.1523/JNEUROSCI.21-19-07543.2001

79. Nematipour S, Vahidinia Z, Nejati M, Naderian H, Beyer C, Azami Tameh A. Estrogen and progesterone attenuate glutamate neurotoxicity via regulation of EAAT3 and GLT-1 in a rat model of ischemic stroke. Iran J Basic Med Sci. Iran; 2020;23:1346–52. https://doi.org/10.22038/ijbms.2020.48090.11039

80. Xu F, Ma R, Zhang G, Wang S, Yin J, Wang E, et al. Estrogen and propofol combination therapy inhibits endoplasmic reticulum stress and remarkably attenuates cerebral ischemia-reperfusion injury and OGD injury in hippocampus. Biomed Pharmacother Biomedecine Pharmacother. France; 2018;108:1596–606. https://doi.org/10.1016/j.biopha.2018.09.167

81. Saleh TM, Connell BJ, Legge C, Cribb AE. Estrogen attenuates neuronal excitability in the insular cortex following middle cerebral artery occlusion. Brain Res. Netherlands; 2004;1018:119–29. https://doi.org/10.1016/j.brainres.2004.05.074

82. Toung TK, Hurn PD, Traystman RJ, Sieber FE. Estrogen decreases infarct size after temporary focal ischemia in a genetic model of type 1 diabetes mellitus. Stroke. United States; 2000;31:2701–6. https://doi.org/10.1161/01.str.31.11.2701

83. Li J, Siegel M, Yuan M, Zeng Z, Finnucan L, Persky R, et al. Estrogen enhances neurogenesis and behavioral recovery after stroke. J Cereb Blood Flow Metab. United States; 2011;31:413–25. https://doi.org/10.1038/jcbfm.2010.181

84. Jia J, Guan D, Zhu W, Alkayed NJ, Wang MM, Hua Z, et al. Estrogen inhibits Fas-mediated apoptosis in experimental stroke. Exp Neurol. United States; 2009;215:48–52. https://doi.org/10.1016/j.expneurol.2008.09.015

85. Saleh MC, Connell BJ, Saleh TM. Estrogen may contribute to ischemic tolerance through modulation of cellular stress-related proteins. Neurosci Res. Ireland; 2009;63:273–9. https://doi.org/10.1016/j.neures.2009.01.004

86. Won C-K, Kim M-O, Koh P-O. Estrogen modulates Bcl-2 family proteins in ischemic brain injury. J Vet Med Sci. Japan; 2006;68:277–80. https://doi.org/10.1292/jvms.68.277

87. Dubal DB, Zhu H, Yu J, Rau SW, Shughrue PJ, Merchenthaler I, et al. Estrogen receptor alpha, not beta, is a critical link in estradiol-mediated protection against brain injury. Proc Natl Acad Sci U A. United States; 2001;98:1952–7. https://doi.org/10.1073/pnas.98.4.1952

88. Nakamura T, Hua Y, Keep R, Park J, Xi G, Hoff J. Estrogen therapy for experimental intracerebral hemorrhage in rats. J Neurosurg. 2005;103:97–103. https://doi.org/10.3171/jns.2005.103.1.0097

89. Su Q., Cheng Y., Jin K., Cheng J., Lin Y., Lin Z., et al. Estrogen therapy increases BDNF expression and improves post-stroke depression in ovariectomy-treated rats. Exp Ther Med. Greece: Spandidos Publications (10 Vriaxidos Street, Athens 116 10, Greece); 2016;12:1843–8. https://doi.org/10.3892/etm.2016.3531

90. Saleh TM, Cribb AE, Connell BJ. Estrogen-induced recovery of autonomic function after middle cerebral artery occlusion in male rats. Am J Physiol Regul Integr Comp Physiol. United States; 2001;281:R1531-9. https://doi.org/10.1152/ajpregu.2001.281.5.R1531

91. Toung TJ, Traystman RJ, Hurn PD. Estrogen-mediated neuroprotection after experimental stroke in male rats. Stroke. United States; 1998;29:1666–70. https://doi.org/10.1161/01.str.29.8.1666

92. Connell BJ, Crosby KM, Richard MJ, Mayne MB, Saleh TM. Estrogen-mediated neuroprotection in the cortex may require NMDA receptor activation. Neuroscience. United States; 2007;146:160–9. https://doi.org/10.1016/j.neuroscience.2007.01.014

93. Shi J, Bui JD, Yang SH, He Z, Lucas TH, Buckley DL, et al. Estrogens decrease reperfusion-associated cortical ischemic damage: an MRI analysis in a transient focal ischemia model. Stroke. United States; 2001;32:987–92. https://doi.org/10.1161/01.str.32.4.987

94. Simpkins JW, Rajakumar G, Zhang YQ, Simpkins CE, Greenwald D, Yu CJ, et al. Estrogens may reduce mortality and ischemic damage caused by middle cerebral artery occlusion in the female rat. J Neurosurg. United States; 1997;87:724–30. https://doi.org/10.3171/jns.1997.87.5.0724

95. Wong R, Gibson CL, Kendall DA, Bath PM. Evaluating the translational potential of progesterone treatment following transient cerebral ischaemia in male mice. BMC Neurosci. England; 2014;15:131. https://doi.org/10.1186/s12868-014-0131-5

96. Chiappetta O, Gliozzi M, Siviglia E, Amantea D, Morrone LA, Berliocchi L, et al. Evidence to implicate early modulation of interleukin-1beta expression in the neuroprotection afforded by 17beta-estradiol in male rats undergone transient middle cerebral artery occlusion. Int Rev Neurobiol. United States; 2007;82:357–72. https://doi.org/10.1016/S0074-7742(07)82019-8

97. Nguyen AP, Arvanitidis AP, Colbourne F. Failure of estradiol to improve spontaneous or rehabilitation-facilitated recovery after hemorrhagic stroke in rats. Brain Res. Netherlands; 2008;1193:109–19. https://doi.org/10.1016/j.brainres.2007.11.054

98. Sakata A, Mogi M, Iwanami J, Tsukuda K, Min LJ, Jing F, et al. Female type 2 diabetes mellitus mice exhibit severe ischemic brain damage. J Am Soc Hypertens. United States; 2011;5:7–11. https://doi.org/10.1016/j.jash.2010.12.003

99. Vergouwen MD, Anderson RE, Meyer FB. Gender differences and the effects of synthetic exogenous and non-synthetic estrogens in focal cerebral ischemia. Brain Res. Netherlands; 2000;878:88–97. https://doi.org/10.1016/s0006-8993(00)02713-x

100. Habib P, Harms J, Zendedel A, Beyer C, Slowik A. Gonadal Hormones E2 and P Mitigate Cerebral Ischemia-Induced Upregulation of the AIM2 and NLRC4 Inflammasomes in Rats. Int J Mol Sci. Switzerland; 2020;21. https://doi.org/10.3390/ijms21134795

101. Vahidinia Z, Alipour N, Atlasi MA, Naderian H, Beyer C, Azami Tameh A. Gonadal steroids block the calpain-1-dependent intrinsic pathway of apoptosis in an experimental rat stroke model. Neurol Res. England; 2017;39:54–64. https://doi.org/10.1080/01616412.2016.1250459

102. Dang J, Mitkari B, Kipp M, Beyer C. Gonadal steroids prevent cell damage and stimulate behavioral recovery after transient middle cerebral artery occlusion in male and female rats. Brain Behav Immun. Netherlands; 2011;25:715–26. https://doi.org/10.1016/j.bbi.2011.01.013

103. Han Z-W, Chang Y-C, Zhou Y, Zhang H, Chen L, Zhang Y, et al. GPER agonist G1 suppresses neuronal apoptosis mediated by endoplasmic reticulum stress after cerebral ischemia/reperfusion injury. Neural Regen Res. 2019;14:1221–9. https://doi.org/10.4103/1673-5374.251571

104. Carpenter RS, Iwuchukwu I, Hinkson CL, Reitz S, Lee W, Kukino A, et al. High-dose estrogen treatment at reperfusion reduces lesion volume and accelerates recovery of sensorimotor function after experimental ischemic stroke. Brain Res. Netherlands; 2016;1639:200–13. https://doi.org/10.1016/j.brainres.2016.01.058

105. Schreihofer DA, Do KD, Schreihofer AM. High-soy diet decreases infarct size after permanent middle cerebral artery occlusion in female rats. Am J Physiol Regul Integr Comp Physiol. United States; 2005;289:R103-8. https://doi.org/10.1152/ajpregu.00642.2004

106. Brown CM, Dela Cruz CD, Yang E, Wise PM. Inducible nitric oxide synthase and estradiol exhibit complementary neuroprotective roles after ischemic brain injury. Exp Neurol. United States; 2008;210:782–7. https://doi.org/10.1016/j.expneurol.2007.11.021

107. Park EM, Cho S, Frys KA, Glickstein SB, Zhou P, Anrather J, et al. Inducible nitric oxide synthase contributes to gender differences in ischemic brain injury. J Cereb Blood Flow Metab. United States; 2006;26:392–401. https://doi.org/10.1038/sj.jcbfm.9600194

108. Stary CM, Xu L, Li L, Sun X, Ouyang Y-B, Xiong X, et al. Inhibition of miR-181a protects female mice from transient focal cerebral ischemia by targeting astrocyte estrogen receptor-α. Mol Cell Neurosci. United States; 2017;82:118–25. https://doi.org/10.1016/j.mcn.2017.05.004

109. Fréchou M, Zhang S, Liere P, Delespierre B, Soyed N, Pianos A, et al. Intranasal delivery of progesterone after transient ischemic stroke decreases mortality and provides neuroprotection. Neuropharmacology. England; 2015;97:394–403. https://doi.org/10.1016/j.neuropharm.2015.06.002

110. Wang L, Wang J, Shan Q, Shu H, Guo J-M. Involvement of baroreflex deficiency in the age-related loss of estrogen efficacy against cerebral ischemia. Front Aging Neurosci. Switzerland; 2023;15:1167170. https://doi.org/10.3389/fnagi.2023.1167170

111. Hayashi S, Ueyama T, Kajimoto T, Yagi K, Kohmura E, Saito N. Involvement of gamma protein kinase C in estrogen-induced neuroprotection against focal brain ischemia through G protein-coupled estrogen receptor. J Neurochem. England; 2005;93:883–91. https://doi.org/10.1111/j.1471-4159.2005.03080.x

112. Yu SJ, Kim JR, Lee CK, Kim JH, Kam KY, Hong JH, et al. Involvement of purα gene in neuroprotection effects of estrogen in rat ischemic brain model. Korean J Genet. 2006;28:403–12.

113. Qin A, Zhang Q, Wang J, Sayeed I, Stein DG. Is a combination of progesterone and chloroquine more effective than either alone in the treatment of cerebral ischemic injury? Restor Neurol Neurosci. Netherlands; 2019;37:1–10. https://doi.org/10.3233/RNN-180837

114. Zhu W, Wang L, Zhang L, Palmateer JM, Libal NL, Hurn PD, et al. Isoflurane preconditioning neuroprotection in experimental focal stroke is androgen-dependent in male mice. Neuroscience. United States; 2010;169:758–69. https://doi.org/10.1016/j.neuroscience.2010.05.038

115. Nguyen T, Su C, Singh M. Let-7i inhibition enhances progesterone-induced functional recovery in a mouse model of ischemia. Proc Natl Acad Sci U A. United States; 2018;115:E9668–77. https://doi.org/10.1073/pnas.1803384115

116. Cordeau PJ, Lalancette-Hébert M, Weng YC, Kriz J. Live imaging of neuroinflammation reveals sex and estrogen effects on astrocyte response to ischemic injury. Stroke. United States; 2008;39:935–42. https://doi.org/10.1161/STROKEAHA.107.501460

117. Ulbrich C, Zendedel A, Habib P, Kipp M, Beyer C, Dang J. Long-term cerebral cortex protection and behavioral stabilization by gonadal steroid hormones after transient focal hypoxia. J Steroid Biochem Mol Biol. England; 2012;131:10–6. https://doi.org/10.1016/j.jsbmb.2012.01.007

118. Oppong-Gyebi A, Metzger D, Doan T, Han J, Vann PH, Yockey RA, et al. Long-term hypogonadism diminishes the neuroprotective effects of dietary genistein in young adult ovariectomized rats after transient focal ischemia. J Neurosci Res. United States; 2022;100:598–619. https://doi.org/10.1002/jnr.24981

119. Amantea D, Spagnuolo P, Bari M, Fezza F, Mazzei C, Tassorelli C, et al. Modulation of the endocannabinoid system by focal brain ischemia in the rat is involved in neuroprotection afforded by 17beta-estradiol. FEBS J. England; 2007;274:4464–775. https://doi.org/10.1111/j.1742-4658.2007.05975.x

120. Scheld M, Heymann F, Zhao W, Tohidnezhad M, Clarner T, Beyer C, et al. Modulatory effect of 17β-estradiol on myeloid cell infiltration into the male rat brain after ischemic stroke. J Steroid Biochem Mol Biol. England; 2020;202:105667. https://doi.org/10.1016/j.jsbmb.2020.105667

121. Chen C, Hu Q, Yan J, Lei J, Qin L, Shi X, et al. Multiple effects of 2ME2 and D609 on the cortical expression of HIF-1alpha and apoptotic genes in a middle cerebral artery occlusion-induced focal ischemia rat model. J Neurochem. England; 2007;102:1831–41. https://doi.org/10.1111/j.1471-4159.2007.04652.x

122. Wang J, Liu M, Hou W, Hou M, Zhang L, Sun M, et al. N-myc Downstream-Regulated Gene 2 (Ndrg2): A Critical Mediator of Estrogen-Induced Neuroprotection Against Cerebral Ischemic Injury. Mol Neurobiol. United States; 2022;59:4793–804. https://doi.org/10.1007/s12035-022-02877-5

123. Persky RW, Liu F, Xu Y, Weston G, Levy S, Roselli CE, et al. Neonatal testosterone exposure protects adult male rats from stroke. Neuroendocrinology. Switzerland; 2013;97:271–82. https://doi.org/10.1159/000343804

124. Tanaka M, Ogaeri T, Samsonov M, Sokabe M. Nestorone exerts long-term neuroprotective effects against transient focal cerebral ischemia in adult male rats. Brain Res. Netherlands; 2019;1719:288–96. https://doi.org/10.1016/j.brainres.2018.09.022

125. Elzer JG, Muhammad S, Wintermantel TM, Regnier-Vigouroux A, Ludwig J, Schütz G, et al. Neuronal estrogen receptor-a mediates neuroprotection by 17Β-estradiol. J Cereb Blood Flow Metab. 2010;30:935–42. https://doi.org/10.1038/jcbfm.2009.258

126. Culmsee C, Vedder H, Ravati A, Junker V, Otto D, Ahlemeyer B, et al. Neuroprotection by estrogens in a mouse model of focal cerebral ischemia and in cultured neurons: evidence for a receptor-independent antioxidative mechanism. J Cereb Blood Flow Metab. United States; 1999;19:1263–9. https://doi.org/10.1097/00004647-199911000-00011

127. Yousuf S, Atif F, Sayeed I, Wang J, Stein DG. Neuroprotection by progesterone after transient cerebral ischemia in stroke-prone spontaneously hypertensive rats. Horm Behav. United States; 2016;84:29–40. https://doi.org/10.1016/j.yhbeh.2016.06.002

128. Kumon Y, Kim SC, Tompkins P, Stevens A, Sakaki S, Loftus CM. Neuroprotective effect of postischemic administration of progesterone in spontaneously hypertensive rats with focal cerebral ischemia. J Neurosurg. United States; 2000;92:848–52. https://doi.org/10.3171/jns.2000.92.5.0848

129. Zhang D, Xia H, Xu L, Zhang C, Yao W, Wang Y, et al. Neuroprotective effects of 17β-estradiol associate with KATP in rat brain. Neuroreport. England; 2012;23:952–7. https://doi.org/10.1097/WNR.0b013e3283598de6

130. Liu R, Yang SH, Perez E, Yi KD, Wu SS, Eberst K, et al. Neuroprotective effects of a novel non-receptor-binding estrogen analogue: in vitro and in vivo analysis. Stroke. United States; 2002;33:2485–91. https://doi.org/10.1161/01.str.0000030317.43597.c8

131. Perez E, Liu R, Yang S-H, Cai ZY, Covey DF, Simpkins JW. Neuroprotective effects of an estratriene analog are estrogen receptor independent in vitro and in vivo. Brain Res. Netherlands; 2005;1038:216–22. https://doi.org/10.1016/j.brainres.2005.01.026

132. Dhote V, Mandloi AS, Singour PK, Kawadkar M, Ganeshpurkar A, Jadhav MP. Neuroprotective effects of combined trimetazidine and progesterone on cerebral reperfusion injury. Curr Res Pharmacol Drug Discov [Internet]. 2022;3. https://doi.org/10.1016/j.crphar.2022.100108

133. Dubal DB, Wise PM. Neuroprotective effects of estradiol in middle-aged female rats. Endocrinology. United States; 2001;142:43–8. https://doi.org/10.1210/endo.142.1.7911

134. Alkayed NJ, Murphy SJ, Traystman RJ, Hurn PD, Miller VM. Neuroprotective effects of female gonadal steroids in reproductively senescent female rats. Stroke. United States; 2000;31:161–8. https://doi.org/10.1161/01.str.31.1.161

135. Chen J, Chopp M, Li Y. Neuroprotective effects of progesterone after transient middle cerebral artery occlusion in rat. J Neurol Sci. Netherlands; 1999;171:24–30. https://doi.org/10.1016/s0022-510x(99)00247-6

136. Wang J, Jiang C, Liu C, Li X, Chen N, Hao Y. Neuroprotective effects of progesterone following stroke in aged rats. Behav Brain Res. Netherlands; 2010;209:119–22. https://doi.org/10.1016/j.bbr.2010.01.026

137. Zhang J, Li H, Xu Z, Lu J, Cao C, Shen H, et al. Oestrogen ameliorates blood-brain barrier damage after experimental subarachnoid haemorrhage via the SHH pathway in male rats. Stroke Vasc Neurol. England; 2023;8:217–28. https://doi.org/10.1136/svn-2022-001907

138. Chang LC, Cheng CJ, Tsai TH, Liu CW, Tsai TR. Optimization of rate-controlled 17beta-estradiol nanoparticles for cerebral ischemia therapy. J Biomed Nanotechnol. United States; 2013;9:1724–35. https://doi.org/10.1166/jbn.2013.1672

139. Fukuda K, Yao H, Ibayashi S, Nakahara T, Uchimura H, Fujishima M, et al. Ovariectomy exacerbates and estrogen replacement attenuates photothrombotic focal ischemic brain injury in rats. Stroke. United States; 2000;31:155–60. https://doi.org/10.1161/01.str.31.1.155

140. Zheng J, Zhang P, Li X, Lei S, Li W, He X, et al. Post-stroke estradiol treatment enhances neurogenesis in the subventricular zone of rats after permanent focal cerebral ischemia. Neuroscience. United States; 2013;231:82–90. https://doi.org/10.1016/j.neuroscience.2012.11.042

141. Stoop W., De Geyter D., Verachtert S., Brouwers S., Verdood P., De Keyser J., et al. Post-stroke treatment with 17beta-estradiol exerts neuroprotective effects in both normotensive and hypertensive rats. Neuroscience. United Kingdom: Elsevier Ltd; 2017;348:335–45. https://doi.org/10.1016/j.neuroscience.2017.02.040

142. McCullough LD, Alkayed NJ, Traystman RJ, Williams MJ, Hurn PD. Postischemic estrogen reduces hypoperfusion and secondary ischemia after experimental stroke. Stroke. United States; 2001;32:796–802. https://doi.org/10.1161/01.str.32.3.796

143. Lammerding L, Slowik A, Johann S, Beyer C, Zendedel A. Poststroke Inflammasome Expression and Regulation in the Peri-Infarct Area by Gonadal Steroids after Transient Focal Ischemia in the Rat Brain. Neuroendocrinology. Switzerland; 2016;103:460–75. https://doi.org/10.1159/000439435

144. Xiao H., Deng M., Yang B., Hu Z., Tang J. Pretreatment with 17beta-Estradiol Attenuates Cerebral Ischemia-Induced Blood-Brain Barrier Disruption in Aged Rats: Involvement of Antioxidant Signaling. Neuroendocrinology. Switzerland: S. Karger AG; 2017;106:20–9. https://doi.org/10.1159/000455866

145. Murphy SJ, Littleton-Kearney MT, Hurn PD. Progesterone administration during reperfusion, but not preischemia alone, reduces injury in ovariectomized rats. J Cereb Blood Flow Metab. United States; 2002;22:1181–8. https://doi.org/10.1097/01.WCB.0000037990.07114.07

146. Wang Z, Zuo G, Shi XY, Zhang J, Fang Q, Chen G. Progesterone administration modulates cortical TLR4/NF-κB signaling pathway after subarachnoid hemorrhage in male rats. Mediat Inflamm. United States; 2011;2011:848309. https://doi.org/10.1155/2011/848309

147. Cai J, Cao S, Chen J, Yan F, Chen G, Dai Y. Progesterone alleviates acute brain injury via reducing apoptosis and oxidative stress in a rat experimental subarachnoid hemorrhage model. Neurosci Lett. Ireland; 2015;600:238–43. https://doi.org/10.1016/j.neulet.2015.06.023

148. Ishrat T, Sayeed I, Atif F, Hua F, Stein DG. Progesterone and allopregnanolone attenuate blood-brain barrier dysfunction following permanent focal ischemia by regulating the expression of matrix metalloproteinases. Exp Neurol. United States; 2010;226:183–90. https://doi.org/10.1016/j.expneurol.2010.08.023

149. Lee RJ, Kim JK, Chao D, Kuo L, Mally A, McClean ME, et al. Progesterone and allopregnanolone improves stroke outcome in male mice via distinct mechanisms but neither promotes neurogenesis. J Neurochem. England; 2015;132:32–7. https://doi.org/10.1111/jnc.12990

150. Yan F, Hu Q, Chen J, Wu C, Gu C, Chen G. Progesterone attenuates early brain injury after subarachnoid hemorrhage in rats. Neurosci Lett. Ireland; 2013;543:163–7. https://doi.org/10.1016/j.neulet.2013.03.005

151. Won S, Lee JH, Wali B, Stein DG, Sayeed I. Progesterone attenuates hemorrhagic transformation after delayed tPA treatment in an experimental model of stroke in rats: involvement of the VEGF-MMP pathway. J Cereb Blood Flow Metab. United States; 2014;34:72–80. https://doi.org/10.1038/jcbfm.2013.163

152. Liu C, Gao W, Zhao L, Cao Y. Progesterone attenuates neurological deficits and exerts a protective effect on damaged axons via the PI3K/AKT/mTOR-dependent pathway in a mouse model of intracerebral hemorrhage. AGING-US. 2022;14:2574–89.

153. Jiang C., Zuo F., Wang Y., Lu H., Yang Q., Wang J. Progesterone Changes VEGF and BDNF Expression and Promotes Neurogenesis After Ischemic Stroke. Mol Neurobiol. United States: Humana Press Inc.; 2017;54:571–81. https://doi.org/10.1007/s12035-015-9651-y

154. Gibson CL, Murphy SP. Progesterone enhances functional recovery after middle cerebral artery occlusion in male mice. J Cereb Blood Flow Metab. United States; 2004;24:805–13. https://doi.org/10.1097/01.WCB.0000125365.83980.00

155. Murphy SJ, Traystman RJ, Hurn PD, Duckles SP. Progesterone exacerbates striatal stroke injury in progesterone-deficient female animals. Stroke. United States; 2000;31:1173–8. https://doi.org/10.1161/01.str.31.5.1173

156. Jiang C, Zuo F, Wang Y, Wan J, Yang Z, Lu H, et al. Progesterone exerts neuroprotective effects and improves long-term neurologic outcome after intracerebral hemorrhage in middle-aged mice. Neurobiol Aging. 2016;42:13–24. https://doi.org/10.1016/j.neurobiolaging.2016.02.029

157. Jiang C, Wang J, Li X, Liu C, Chen N, Hao Y. Progesterone exerts neuroprotective effects by inhibiting inflammatory response after stroke. Inflamm Res. Switzerland; 2009;58:619–24. https://doi.org/10.1007/s00011-009-0032-8

158. Tanaka M, Ogaeri T, Samsonov M, Sokabe M. Progesterone improves functional outcomes after transient focal cerebral ischemia in both aged male and female rats. Exp Gerontol. England; 2018;113:29–35. https://doi.org/10.1016/j.exger.2018.09.012

159. Wali B, Ishrat T, Stein DG, Sayeed I. Progesterone improves long-term functional and histological outcomes after permanent stroke in older rats. Behav Brain Res. Netherlands; 2016;305:46–56. https://doi.org/10.1016/j.bbr.2016.02.024

160. Lei B, Wang H, Jeong S, Hsieh JT, Majeed M, Dawson H, et al. Progesterone Improves Neurobehavioral Outcome in Models of Intracerebral Hemorrhage. NEUROENDOCRINOLOGY. 2016;103:665–77. https://doi.org/10.1159/000442204

161. Wali B, Ishrat T, Won S, Stein DG, Sayeed I. Progesterone in experimental permanent stroke: a dose-response and therapeutic time-window study. Brain. England; 2014;137:486–502. https://doi.org/10.1093/brain/awt319

162. Yousuf S, Atif F, Sayeed I, Tang H, Stein DG. Progesterone in transient ischemic stroke: a dose-response study. Psychopharmacol Berl. Germany; 2014;231:3313–23. https://doi.org/10.1007/s00213-014-3556-8

163. Andrabi SS, Parvez S, Tabassum H. Progesterone induces neuroprotection following reperfusion-promoted mitochondrial dysfunction after focal cerebral ischemia in rats. Model Mech. England; 2017;10:787–96. https://doi.org/10.1242/dmm.025692

164. Wang J, Zhao Y, Liu C, Jiang C, Zhao C, Zhu Z. Progesterone inhibits inflammatory response pathways after permanent middle cerebral artery occlusion in rats. Mol Med Rep. Greece; 2011;4:319–24. https://doi.org/10.3892/mmr.2011.418

165. Sayeed I, Wali B, Stein DG. Progesterone inhibits ischemic brain injury in a rat model of permanent middle cerebral artery occlusion. Restor Neurol Neurosci. Netherlands; 2007;25:151–9.

166. Jiang N, Chopp M, Stein D, Feit H. Progesterone is neuroprotective after transient middle cerebral artery occlusion in male rats. Brain Res. Netherlands; 1996;735:101–7. https://doi.org/10.1016/0006-8993(96)00605-1

167. Ishrat T, Sayeed I, Atif F, Hua F, Stein DG. Progesterone is neuroprotective against ischemic brain injury through its effects on the phosphoinositide 3-kinase/protein kinase B signaling pathway. Neuroscience. United States; 2012;210:442–50. https://doi.org/10.1016/j.neuroscience.2012.03.008

168. Gibson CL, Coomber B, Murphy SP. Progesterone is neuroprotective following cerebral ischaemia in reproductively ageing female mice. Brain. England; 2011;134:2125–33. https://doi.org/10.1093/brain/awr132

169. Tanaka M, Sokabe M, Asai M. Progesterone Receptor Agonist, Nestorone, Exerts Long-Term Neuroprotective Effects Against Permanent Focal Cerebral Ischemia in Adult and Aged Male Rats. Transl Stroke Res. United States; 2024; https://doi.org/10.1007/s12975-024-01288-z

170. Liu A, Margaill I, Zhang S, Labombarda F, Coqueran B, Delespierre B, et al. Progesterone receptors: a key for neuroprotection in experimental stroke. Endocrinology. United States; 2012;153:3747–57. https://doi.org/10.1210/en.2012-1138

171. Gibson CL, Constantin D, Prior MJ, Bath PM, Murphy SP. Progesterone suppresses the inflammatory response and nitric oxide synthase-2 expression following cerebral ischemia. Exp Neurol. United States; 2005;193:522–30. https://doi.org/10.1016/j.expneurol.2005.01.009

172. Allen RS, Olsen TW, Sayeed I, Cale HA, Morrison KC, Oumarbaeva Y, et al. Progesterone treatment in two rat models of ocular ischemia. Invest Ophthalmol Vis Sci. United States; 2015;56:2880–91. https://doi.org/10.1167/iovs.14-16070

173. Glendenning ML, Lovekamp-Swan T, Schreihofer DA. Protective effect of estrogen in endothelin-induced middle cerebral artery occlusion in female rats. Neurosci Lett. Ireland; 2008;445:188–92. https://doi.org/10.1016/j.neulet.2008.09.006

174. Liu JJ, Pan SY. Protective effects of estrogen combined with sevoflurane in an experimental model of cerebral infarction and focal cerebral ischemia-reperfusion injury. Eur Rev Med Pharmacol Sci. Italy; 2016;20:1839–44.

175. Alimohamadi R., Fatemi I., Naderi S., Hakimizadeh E., Rahmani M.-R., Allahtavakoli M. Protective effects of Vitex agnus-castus in ovariectomy mice following permanent middle cerebral artery occlusion. Iran J Basic Med Sci. Iran, Islamic Republic of: Mashhad University of Medical Sciences (P.O. Box: 445, Daneshgah Avenue, Mashhad, Iran, Islamic Republic of. E-mail: ijp@mums.ac.ir); 2019;22:1097–101. https://doi.org/10.22038/ijbms.2019.31692.7625

176. Prokai L, Prokai-Tatrai K, Perjesi P, Zharikova AD, Perez EJ, Liu R, et al. Quinol-based cyclic antioxidant mechanism in estrogen neuroprotection. Proc Natl Acad Sci U S A. 2003;100:11741–6. https://doi.org/10.1073/pnas.2032621100

177. Saleh TM, Cribb AE, Connell BJ. Reduction in infarct size by local estrogen does not prevent autonomic dysfunction after stroke. Am J Physiol Regul Integr Comp Physiol. United States; 2001;281:R2088-95. https://doi.org/10.1152/ajpregu.2001.281.6.R2088

178. Selvamani A, Sohrabji F. Reproductive age modulates the impact of focal ischemia on the forebrain as well as the effects of estrogen treatment in female rats. Neurobiol Aging. United States; 2010;31:1618–28. https://doi.org/10.1016/j.neurobiolaging.2008.08.014

179. Park MJ, Pilla R, Panta A, Pandey S, Sarawichitr B, Suchodolski J, et al. Reproductive Senescence and Ischemic Stroke Remodel the Gut Microbiome and Modulate the Effects of Estrogen Treatment in Female Rats. Transl Stroke Res. United States; 2020;11:812–30. https://doi.org/10.1007/s12975-019-00760-5

180. Dziennis S, Akiyoshi K, Subramanian S, Offner H, Hurn PD. Role of dihydrotestosterone in post-stroke peripheral immunosuppression after cerebral ischemia. Brain Behav Immun. Netherlands; 2011;25:685–95. https://doi.org/10.1016/j.bbi.2011.01.009

181. Cheng J, Uchida M, Zhang W, Grafe MR, Herson PS, Hurn PD. Role of salt-induced kinase 1 in androgen neuroprotection against cerebral ischemia. J Cereb Blood Flow Metab. United States; 2011;31:339–50. https://doi.org/10.1038/jcbfm.2010.98

182. Dziennis S, Jia T, Rønnekleiv OK, Hurn PD, Alkayed NJ. Role of signal transducer and activator of transcription-3 in estradiol-mediated neuroprotection. J Neurosci Off J Soc Neurosci. United States; 2007;27:7268–74. https://doi.org/10.1523/JNEUROSCI.1558-07.2007

183. Tameh AA, Karimian M, Zare-Dehghanani Z, Aftabi Y, Beyer C. Role of Steroid Therapy after Ischemic Stroke by n-Methyl-d-Aspartate Receptor Gene Regulation. J Stroke Cerebrovasc Dis. United States; 2018;27:3066–75. https://doi.org/10.1016/j.jstrokecerebrovasdis.2018.06.041

184. Liu H, Zhong L, Zhang Y, Liu X, Li J. Rutin attenuates cerebral ischemia-reperfusion injury in ovariectomized rats via estrogen-receptor-mediated BDNF-TrkB and NGF-TrkA signaling. Biochem Cell Biol Biochim Biol Cell. Canada; 2018;96:672–81. https://doi.org/10.1139/bcb-2017-0209

185. Selvaraj UM, Zuurbier KR, Whoolery CW, Plautz EJ, Chambliss KL, Kong X, et al. Selective Nonnuclear Estrogen Receptor Activation Decreases Stroke Severity and Promotes Functional Recovery in Female Mice. Endocrinology. United States; 2018;159:3848–59. https://doi.org/10.1210/en.2018-00600

186. Manwani B, Bentivegna K, Benashski SE, Venna VR, Xu Y, Arnold AP, et al. Sex differences in ischemic stroke sensitivity are influenced by gonadal hormones, not by sex chromosome complement. J Cereb Blood Flow Metab. United States; 2015;35:221–9. https://doi.org/10.1038/jcbfm.2014.186

187. Fréchou M, Zhu X, Kumar N, Sitruk-Ware R, Schumacher M, Mattern C, et al. Sex differences in the cerebroprotection by Nestorone intranasal delivery following stroke in mice. Neuropharmacology. England; 2021;198:108760. https://doi.org/10.1016/j.neuropharm.2021.108760

188. Quillinan N, Grewal H, Klawitter J, Herson PS. Sex Steroids Do Not Modulate TRPM2-Mediated Injury in Females following Middle Cerebral Artery Occlusion(1,2,3). eNeuro. 2014;1. https://doi.org/10.1523/ENEURO.0022-14.2014

189. Patrizz AN, Moruno-Manchon JF, O’Keefe LM, Doran SJ, Patel AR, Venna VR, et al. Sex-Specific Differences in Autophagic Responses to Experimental Ischemic Stroke. Cells. Switzerland; 2021;10. https://doi.org/10.3390/cells10071825

190. Hsieh J.T., Lei B., Sheng H., Venkatraman T., Lascola C.D., Warner D.S., et al. Sex-specific effects of progesterone on early outcome of intracerebral hemorrhage. Neuroendocrinology. Switzerland: S. Karger AG; 2016;103:518–30. https://doi.org/10.1159/000440883

191. Diaz F, Raval AP. Simultaneous nicotine and oral contraceptive exposure alters brain energy metabolism and exacerbates ischemic stroke injury in female rats. J Cereb Blood Flow Metab. 2021;41:793–804. https://doi.org/10.1177/0271678X20925164

192. Joachim E, Barakat R, Lew B, Kim KK, Ko C, Choi H. Single intranasal administration of 17β-estradiol loaded gelatin nanoparticles confers neuroprotection in the post-ischemic brain. Nanomedicine Nanotechnol Biol Med. United States; 2020;29:102246. https://doi.org/10.1016/j.nano.2020.102246

193. Guo JM, Shu H, Wang L, Xu JJ, Niu XC, Zhang L. SIRT1-dependent AMPK pathway in the protection of estrogen against ischemic brain injury. CNS Neurosci Ther. England; 2017;23:360–9. https://doi.org/10.1111/cns.12686

194. Coomber B, Gibson CL. Sustained levels of progesterone prior to the onset of cerebral ischemia are not beneficial to female mice. Brain Res. Netherlands; 2010;1361:124–32. https://doi.org/10.1016/j.brainres.2010.09.037

195. Patkar S., Uwanogho D., Modo M., Tate R.J., Plevin R., Carswell H.V.O. Targeting 17beta-estradiol biosynthesis in neural stem cells improves stroke outcome. Front Cell Neurosci. Switzerland: Frontiers Media SA; 2022;16:917181. https://doi.org/10.3389/fncel.2022.917181

196. Fanaei H, Karimian SM, Sadeghipour HR, Hassanzade G, Kasaeian A, Attari F, et al. Testosterone enhances functional recovery after stroke through promotion of antioxidant defenses, BDNF levels and neurogenesis in male rats. Brain Res. Netherlands; 2014;1558:74–83. https://doi.org/10.1016/j.brainres.2014.02.028

197. Naderi S, Alimohammadi R, Hakimizadeh E, Roohbakhsh A, Shamsizadeh A, Allahtavakoli M. The effect of exercise preconditioning on stroke outcome in ovariectomized mice with permanent middle cerebral artery occlusion. Can J Physiol Pharmacol. Canada; 2018;96:287–94. https://doi.org/10.1139/cjpp-2017-0157

198. Vahidinia Z, Mahdavi E, Talaei SA, Naderian H, Tamtaji A, Haddad Kashani H, et al. The effect of female sex hormones on Hsp27 phosphorylation and histological changes in prefrontal cortex after tMCAO. Pathol Res Pr. Germany; 2021;221:153415. https://doi.org/10.1016/j.prp.2021.153415

199. Ma Y-L, Qin P, Li Y, Shen L, Wang S-Q, Dong H-L, et al. The effects of different doses of estradiol (E2) on cerebral ischemia in an in vitro model of oxygen and glucose deprivation and reperfusion and in a rat model of middle carotid artery occlusion. BMC Neurosci. England; 2013;14:118. https://doi.org/10.1186/1471-2202-14-118

200. Cai M, Ma YL, Qin P, Li Y, Zhang LX, Nie H, et al. The loss of estrogen efficacy against cerebral ischemia in aged postmenopausal female mice. Neurosci Lett. Ireland; 2014;558:115–9. https://doi.org/10.1016/j.neulet.2013.11.007

201. Gröger M, Plesnila N. The neuroprotective effect of 17β-estradiol is independent of its antioxidative properties. Brain Res. Netherlands; 2014;1589:61–7. https://doi.org/10.1016/j.brainres.2014.08.029

202. Selvamani A, Sohrabji F. The neurotoxic effects of estrogen on ischemic stroke in older female rats is associated with age-dependent loss of insulin-like growth factor-1. J Neurosci Off J Soc Neurosci. United States; 2010;30:6852–61. https://doi.org/10.1523/JNEUROSCI.0761-10.2010

203. Green PS, Yang SH, Nilsson KR, Kumar AS, Covey DF, Simpkins JW. The nonfeminizing enantiomer of 17beta-estradiol exerts protective effects in neuronal cultures and a rat model of cerebral ischemia. Endocrinology. United States; 2001;142:400–6. https://doi.org/10.1210/endo.142.1.7888

204. Zhang Z, Qin P, Deng Y, Ma Z, Guo H, Guo H, et al. The novel estrogenic receptor GPR30 alleviates ischemic injury by inhibiting TLR4-mediated microglial inflammation. J Neuroinflammation. England; 2018;15:206. https://doi.org/10.1186/s12974-018-1246-x

205. Liang CC, Liu HL, Chang SD, Chen SH, Lee TH. The Protective Effect of Human Umbilical Cord Blood CD34+ Cells and Estradiol against Focal Cerebral Ischemia in Female Ovariectomized Rat: Cerebral MR Imaging and Immunohistochemical Study. PLoS One. United States; 2016;11:e0147133. https://doi.org/10.1371/journal.pone.0147133

206. Castelló-Ruiz M, Torregrosa G, Burguete MC, Miranda FJ, Centeno JM, López-Morales MA, et al. The selective estrogen receptor modulator, bazedoxifene, reduces ischemic brain damage in male rat. Neurosci Lett. 2014;575:53–7. https://doi.org/10.1016/j.neulet.2014.05.024

207. Burguete MC, Jover-Mengual T, López-Morales MA, Aliena-Valero A, Jorques M, Torregrosa G, et al. The selective oestrogen receptor modulator, bazedoxifene, mimics the neuroprotective effect of 17B-oestradiol in diabetic ischaemic stroke by modulating oestrogen receptor expression and the MAPK/ERK1/2 signalling pathway. J Neuroendocrinol. United States; 2019;31:e12751. https://doi.org/10.1111/jne.12751

208. Yang S-H, Liu R, Wu SS, Simpkins JW. The use of estrogens and related compounds in the treatment of damage from cerebral ischemia. Ann N Y Acad Sci. United States; 2003;1007:101–7. https://doi.org/10.1196/annals.1286.010

209. Suzuki S, Brown CM, Dela Cruz CD, Yang E, Bridwell DA, Wise PM. Timing of estrogen therapy after ovariectomy dictates the efficacy of its neuroprotective and antiinflammatory actions. Proc Natl Acad Sci U A. United States; 2007;104:6013–8. https://doi.org/10.1073/pnas.0610394104

210. Leon RL, Li X, Huber JD, Rosen CL. Worsened outcome from middle cerebral artery occlusion in aged rats receiving 17β-estradiol. Endocrinology. United States; 2012;153:3386–93. https://doi.org/10.1210/en.2011-1859

211. Turtzo LC, Siegel C, McCullough LD. X chromosome dosage and the response to cerebral ischemia. J Neurosci Off J Soc Neurosci. United States; 2011;31:13255–9. https://doi.org/10.1523/JNEUROSCI.0621-11.2011

212. Atif F, Yousuf S, Espinosa-Garcia C, Harris WAC, Stein DG. Post-ischemic stroke systemic inflammation: Immunomodulation by progesterone and vitamin D hormone. Neuropharmacology. England; 2020;181:108327. https://doi.org/10.1016/j.neuropharm.2020.108327

213. Yousuf S, Atif F, Sayeed I, Wang J, Stein DG. Post-stroke infections exacerbate ischemic brain injury in middle-aged rats: immunomodulation and neuroprotection by progesterone. Neuroscience. United States; 2013;239:92–102. https://doi.org/10.1016/j.neuroscience.2012.10.017

214. Wilson BC, Connell B, Saleh TM. Relaxin-induced reduction of infarct size in male rats receiving MCAO is dependent on nitric oxide synthesis and not estrogenic mechanisms. Neurosci Lett. Ireland; 2006;393:160–4. https://doi.org/10.1016/j.neulet.2005.09.059

215. Nakagawa S, Yang Y, Macartney EL, Spake R, Lagisz M. Quantitative evidence synthesis: a practical guide on meta-analysis, meta-regression, and publication bias tests for environmental sciences. Environ Evid. 2023;12:8. https://doi.org/10.1186/s13750-023-00301-6

216. Bartoń K. MuMIn: Multi-Model Inference [Internet]. 2024. https://CRAN.R-project.org/package=MuMIn

217. Stroke Therapy Academic Industry Roundtable (STAIR). Recommendations for Standards Regarding Preclinical Neuroprotective and Restorative Drug Development. Stroke J Cereb Circ. 1999;30:2752–8. https://doi.org/10.1161/01.STR.30.12.2752

218. Fisher M, Feuerstein G, Howells DW, Hurn PD, Kent TA, Savitz SI. Update of the Stroke Therapy Academic Industry Roundtable Preclinical Recommendations. Stroke J Cereb Circ. 2009;40:2244–50. https://doi.org/10.1161/STROKEAHA.108.541128

219. Lyden P, Buchan A, Boltze J, Fisher M, Ansari S, Broderick JP, et al. Top Priorities for Cerebroprotective Studies—A Paradigm Shift: Report From STAIR XI. Stroke J Cereb Circ. 2021;52:3063–71. https://doi.org/10.1161/STROKEAHA.121.034947

220. Krauth D, Woodruff TJ, Bero L. Instruments for Assessing Risk of Bias and Other Methodological Criteria of Published Animal Studies: A Systematic Review. Environ Health Perspect. 2013;121:985–92. https://doi.org/10.1289/ehp.1206389
